# ScaHybNet: a scalogram-based hybrid ensemble network for ECG arrhythmia classification

**DOI:** 10.1038/s41598-026-53755-2

**Published:** 2026-06-03

**Authors:** Sonam Nagar, Karan Verma, Sachin Singh, Kumar Shashvat

**Affiliations:** 1 National Institute of Technology, Delhi, New Delhi India; 2 Manipal University Jaipur, Jaipur, India

**Keywords:** Arrhythmia classification, Continuous wavelet transform, Scalogram imaging, Transformer architecture, Multi-head self-attention, ScaHybNet, Cardiology, Computational biology and bioinformatics, Engineering, Mathematics and computing, Medical research

## Abstract

Cardiovascular diseases are the leading cause of death in the world, requiring the accurate and timely detection of arrhythmias to prevent sudden cardiac death. In this work, ScaHybNet, a deep learning ensemble model is proposed for multi-class arrhythmia classification using the widely adopted ECG Heartbeat Categorization Dataset. The dataset comprises 109,446 samples across five heartbeat classes (N, S, V, F, Q), enabling comprehensive arrhythmia analysis. The proposed method first transforms the ECG signals to 224 × 224 RGB-scalogram images using CWT with the Morlet wavelet. Then, a hybrid model is developed, which is composed of (1) a residual block-based CNN with skip connections to learn spatial features, (2) a BiLSTM layer for learning temporal features from the CNN feature maps and (3) a Transformer encoder layer with a custom-built multi-head self-attention mechanism to capture long-term dependencies. Thus, to address the extreme class imbalance within the data, stratified balancing of the data among normal beat, supraventricular ectopic beat, ventricular ectopic beat, fusion beat, and unknown beat, and inverse-frequency class weighting were performed. They assessed model robustness using fivefold cross-validation. Hyperparameters set to final values included a batch size of 2, 150 epochs, and an Adam optimizer. Ensemble train accuracy 99.81% and the mean accuracy on the fivefold cross validation set was 90.42% ± 1.26 (std) for ScaHybNet. On the test set (unseen data), it showed a total ensemble test accuracy of 94.73%, precision of 76.51%, recall of 82.93%, and F1-score of 77.40%. The ablation test proved the joint efficacy of each part of the model, and state-of-the-art analysis revealed better or equal results on current standards regarding ECG data with noise and imbalance. ScaHybNet appears to offer the potential to act as a more patient-centric tool that could offer considerable benefits to the medical field.

## Introduction

Arrhythmia is a cardiac rhythm disorder characterized by irregular, excessively fast (tachycardia), slow (bradycardia), or abnormal heartbeat patterns, and is strongly associated with cardiovascular diseases, often leading to severe outcomes such as sudden cardiac death, hemodynamic collapse, and cardiac arrest^1^. Among these, atrial fibrillation (AF) is the most prevalent and clinically significant arrhythmia, accounting for the majority of sustained arrhythmias worldwide, with epidemiological studies indicating that nearly one in three individuals will develop a potentially life-threatening rhythm disorder during their lifetime^2^, alongside a steep rise in global AF incidence and prevalence^3^.

**Fig. 1:**
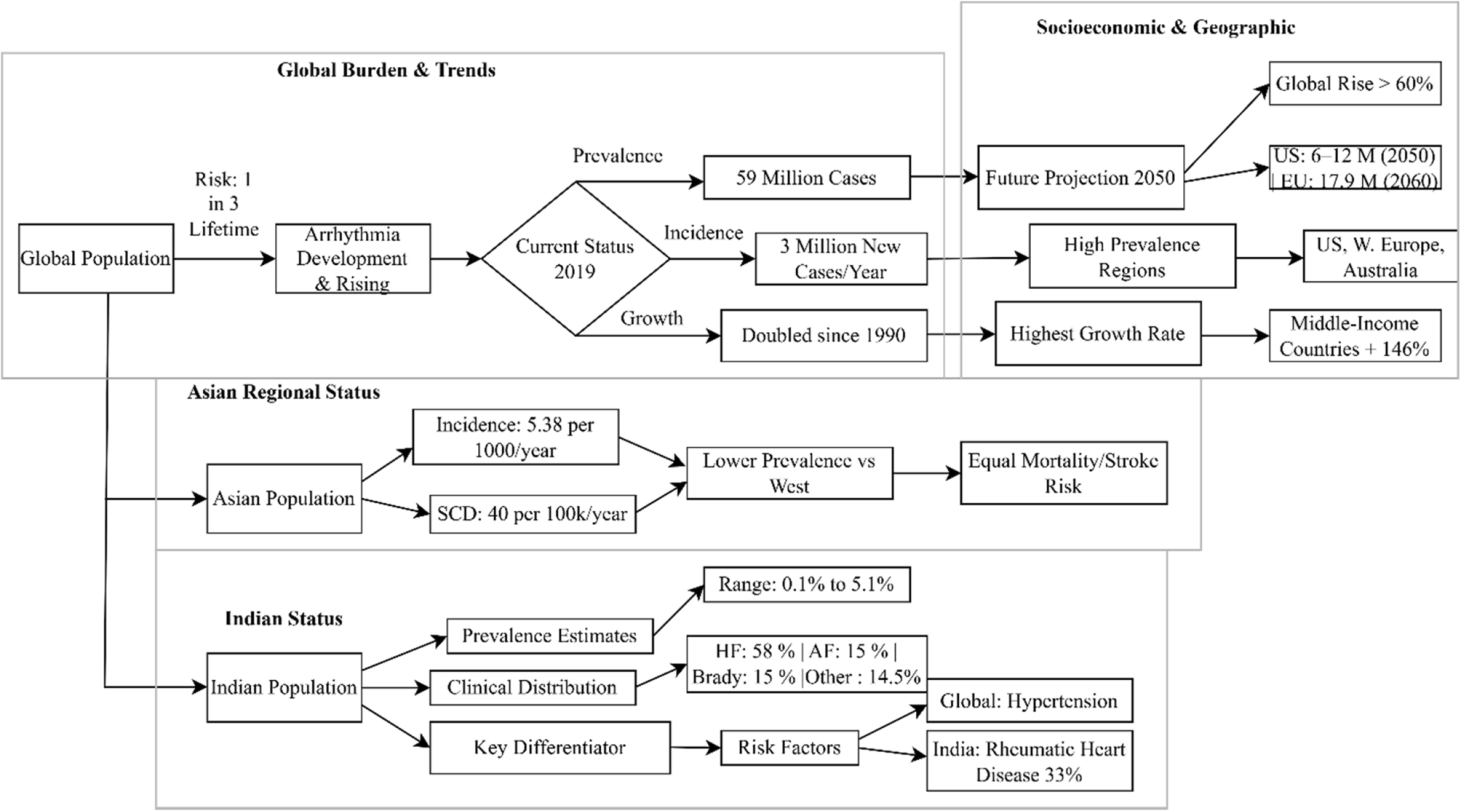
Shows the epidemiological burden of cardiac arrhythmias in the world.

**Fig. 2:**
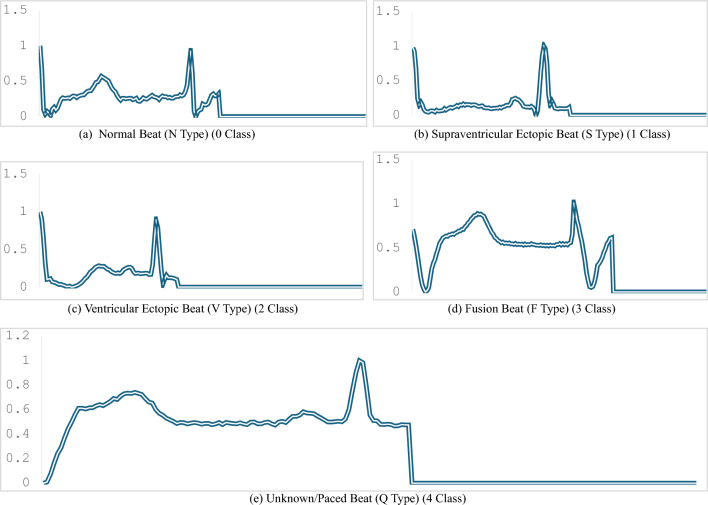
Arrhythmia beat types.

**Fig. 3:**
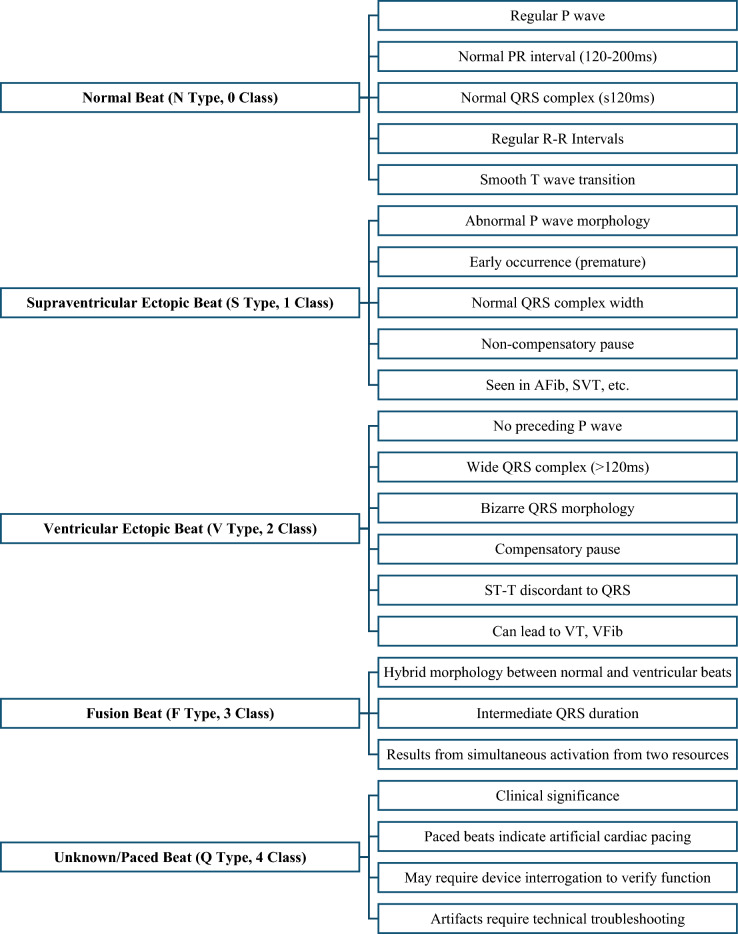
Arrhythmia beats morphology characteristics^15^.

While the level of heterogeneity seen across multiple geographic regions, particularly within Asia and India^4–11^, adds to the complexity and scale of this worldwide health concern, as depicted in Fig. 1^2,3,12–14^, there has been divergence in research and development related to how best to diagnose cardiac arrhythmia. Although the ECG remains the most commonly used non-invasive clinical technique to diagnose arrhythmia, interpretation of the ECG is labor-intensive, is subject to practitioner-to-practitioner variability, is not appropriate for use for mass screening procedures or for the purposes of extended diagnostic monitoring, such as 24-h Holter monitoring, which has been shown to result in over 100,000 ECG records being collected from each patient. Because of this, the MIT-BIH Arrhythmia Database, comprising 48 annotated two-lead ECG recordings and representing five standard heart rhythm categories (i.e., N, S, V, F, Q), is widely recognized as a benchmark dataset for the development of automated arrhythmia detection systems. Due to the large diversity in waveform morphology and temporal features among these categories (as can be observed in Figs. 2, 3^15^), powerful multi-scale spatial and temporal features extraction techniques should be utilized. Regarding the data sources for heartbeat classification in this study, the ECG Heartbeat Categorization Dataset (on Kaggle) was used which contains heartbeat signals from two standard sources, i.e., MIT-BIH Arrhythmia Database and PTB Diagnostic ECG Database. The high amount of data in the ECG Heartbeat Categorization Dataset makes it appropriate for deep learning model training and it is heavily used in studies about heartbeat classification by deep neural networks and validation of transfer learning strategies. It contains ECG signals (normal condition and pathologies like arrhythmia, myocardial infarction) segmented into individual heartbeats after the signals were pre-processed and segmented appropriately. Here the dataset combining data from MIT-BIH and PTB is used to build the model and further for validation across dataset using PTB for model generalization test. Additionally, performance evaluation is conducted across multiple datasets, and the results are systematically analyzed and presented through graphical representations to facilitate comprehensive comparative analysis. Currently machine learning and even more recently deep learning algorithms have shown great promise in performing a fully automated analysis of ECG records and offering clinical decision support at scale and at real time without necessitating human interpretation of the records. CNNs have taken the lead in this regard and have used their capacity to analyze spatial and morphological features to train machines to distinguish between the standard ECG patterns and the various cardiac arrhythmias (using residuals, attention mechanisms, etc.) and representing 60% of all of the deep learning studies of the sort shown as^16–19^, and many more are found between 2020 and 2026. Hybrid CNN-RNN models such as CNN-LSTM and CNN-BiLSTM have emerged as the dominant architectures for ECG analysis. These systems combine spatial feature learning with temporal dependency modelling and are capable of achieving accuracies between 98–99%. On the horizon are transformer models, which use self-attention mechanisms to enable the extraction of long-term temporal dependencies, removing the need to detect R-peaks explicitly. Hybrid CNN-Transformer systems have been recognized as the current leader in ECG analysis due to their ability to capture both local morphology and global rhythm information. Additionally, there is a growing interest in using ensemble learning and optimization methods to enhance the robustness, generalizability and clinical validity of ECG analysis. Specifically, there is evidence that gradient boosting, meta-learning, and validation-aware fusion models can improve the diagnostic performance of ECG analysis systems that exhibit high levels of class imbalance or inter-patient variability. Notwithstanding the advancements made, there are still barriers to achieving noise-resilient, robust, physiological generalizable, interpretable and deployable systems in real world clinical settings with limited resources. Mainly, in this work, we are proposing a hybrid deep learning framework that combines the modeling of spatial, temporal and global dependency for an effective ECG classification. In this evolving environment we present ScaHybNet, a continuous wavelet transforms (CWT) based triadic hybrid ensemble driven by residual CNNs, BiLSTMs and custom-designed multi-head attention Transformers that capture complementary spatial, temporal, and global dependencies in human ECG signals through physiological knowledge infusion. Unlike conventional CNN-RNN or CNN-Transformer models, the proposed framework jointly models spatial, temporal, and global dependencies within a unified architecture. By exploiting CWT-based scalogram feature representations to improve feature robustness and eliminate the need for manual feature engineering, and by using validation-weighted softmax fusion to adaptively combine predictions from different models, ScaHybNet demonstrates improved classification accuracy (1.2–2.3% relative improvement over individual models), robust convergence through increased training patience, and guaranteed generalizability for diverse patient populations. Prioritizing interpretability, computational efficiency, and ethical deployment, the proposed approach significantly advances automated, human-centric ECG arrhythmia classification for five heartbeat classes on the ECG Heartbeat ECG Heartbeat Categorization Dataset, MIT-BIH Arrhythmia Database^20,21^, with high potential for real-world clinical decision support and large-scale cardiovascular risk reduction. The proposed system is designed for large-scale, real-time, and clinical meaningful interpretation of ECG signals for the resource constrained medical facilities. The following paragraph gives a deeper detail on the rationale behind the system design and the practical challenges involved: Recent findings indicate that state-of-art optimization methods combined with hybrid deep learning architectures are efficient for the detection and diagnosis of cardiovascular diseases from ECG signals. Prior research has explored dense neural network architectures combined with optimization strategies to enhance predictive accuracy and convergence behavior. Similarly, hybrid learning frameworks integrating feature selection, ensemble learning, and optimization-based approaches have shown improved robustness in handling complex and imbalanced biomedical datasets. In addition, hybrid CNN-recurrent architectures and attention-based models have significantly improved the ability to capture both spatial and temporal dependencies in ECG signals. These advancements emphasize the importance of integrating optimization strategies and hybrid learning paradigms, thereby motivating the design of the proposed ScaHybNet framework and to overcome the limitations of current methods for ECG arrhythmia detection, we have created a framework to provide clinicians with a reliable tool. The main goal of this framework is to integrate learnings from space, temporal, contextual using a mixed-use structure combining CWT-derived scalogram learning along with residual CNN, bi-directional LSTM and network-type neural architectures called Transforms. Rather than applying time–frequency as an inherent preprocessing step, we have taken CWT representations and made them part of an adaptive diagnostic space that will allow cross- scale features to be learned regardless of noise, and variability between patients. To mitigate instability in ensemble predictions, we have created a Softmax validation-weighted method that has done away with traditional methods of static averages for aggregating vote contribution from each network by using varying weights to adjust each model’s contribution based upon its performance. The emphasis of this framework is practical clinical application, and therefore no synthetic data will be generated through augmentation as we will ensure that meeting physiological authenticity, interpretability, and ethical principles for AI will be priority number one. In summary, this framework will aid in closing the gap between high laboratory accuracy rates of ECG arrhythmia detection with actual real-life clinical efficacy to develop robust, interpretable, scalable systems of classification into arrhythmias thereby providing clinicians with support for reliable decision-making to numerous patients across varying populations.

### Major key contributions

- Adaptive Cross-Scale CWT Feature Representation: The proposed method presents an adaptive scalogram representation based on CWT that enables cross-scale feature learning from human ECG signals. The scalogram representation enhances the robustness of the method against noise and inter-patient variability without requiring feature engineering.
- Residual Morphology Encoder for ECG Analysis: A physiologically informed residual convolutional neural network is proposed to represent ECG morphology and maintain waveform information regardless of the network depth. Residual learning improves the ability to retain diagnostically important information, such as QRS morphology and ST-T changes.
- Feature-to-Sequence Temporal Reprojection Mechanism: A feature-to-sequence reprojection approach is proposed to represent high-level CNN features as sequences of information. This allows recurrent models to capture the evolution of rhythm and temporal relationships between beats at a semantic level of representation.
- Context-Aware Transformer for Long-Range Modeling: We propose a customizable multi-head self-attention transformer, which allows for capturing long-term temporal dependencies and eliminates the need for R-peak detection. The proposed self-attention mechanism specifically emphasizes clinically meaningful temporal features and exploits their potential to increase sensitivity to complex rhythm patterns.
- Validation-Weighted Softmax Ensemble Fusion: A method of combining the input of different types of deep learning models using Validation-Weighted Softmax Fusion has been developed. Cross Validation data will be used to create new prediction algorithms for Improved Classification Stability as well as consistent performance enhancements over individual model usage.
- Clinically Oriented and Human-Centric Design: Since the entire system has been developed primarily for use as a clinical tool, it does not include any form or type of synthetic data augmentation or augmentation method, nor does it perform validation-aware training methods. Therefore, the overall physiological realism of the architecture is preserved through this process.

### Manuscript organization

This paper consists of the following main sections: Section "Introduction" and Section "Literature work" describe background information and give a brief review on the key studies on ECG arrhythmia classification using machine learning and deep learning methods. Section "Proposed ScaHybNet framework pipeline" explains the architecture of Scalable Hybrid Network and details some information regarding data preprocessing, scalogram generation using CWT and a schema of the experiment. Section "Bi-LSTM temporal modeling" presents experimental results and findings and compares them with published research demonstrating the state-of-the-art. Section "Custom Multi-Head Self-Attention (Deep Formulation)" concludes the paper and discuss about future research of ECG arrhythmia classification.

## Literature work

Ensemble learning techniques are becoming increasingly effective for arrhythmia detection, combining multiple models to achieve higher classification performance than individual classifiers, while also addressing problems such as class imbalance, overfitting, and generalization. Mandala et al.^22^ proposes the AD-Ensemble SVM-NB-RF method demonstrated 44.57%, 52.41%, and 29.49% higher accuracy compared with existing models including multi-model ensemble of deep learning for ECG heartbeats arrhythmia categorization (AD-Ensemble CNN-LSTM-RRHOS), VGGNet-based neural network classification (AD-Ensemble CNN-LSTM), and ensemble learning with PSD-based feature extraction (AD-Ensemble MLP-NB-RF). Shao et al.^23^ presents a traditional machine learning ensemble methods have shown remarkable efficacy, with Fine Tuned Boosting (FTBO) model based on boosting algorithm achieving 100% sensitivity and specificity for Atrial Fibrillation detection, 99% sensitivity and specificity for Premature Ventricular Contraction detection in both leads, and almost 96% sensitivity and specificity for Atrial Premature Contraction detection when utilizing a sliding window with window size of 5 R-peaks for feature extraction from multi-lead ECG data. Glaser et al.^24^ made a advanced techniques based on Gradient boosting have been shown to work exceptionally well; through systematic reviews, CatBoost and SVM (Support Vector Machine) were found to be effective and feasible for prediction and detection of new-onset atrial fibrillation (AF) in ICU patients. Pławiak et al.^25^ proposed by using CatBoost with 17 features, an overall F1 score of 0.92 was achieved from the test set (7,270 samples) used for wearable ECG-based detection of AF. Zhao et al.^26^ proposed the integration of deep learning and ensemble approaches has produced promising results; a three-layer deep genetic ensemble classifier (DGEC) that integrates ensemble learning, deep learning, and evolutionarily based computations achieved sensitivity (94.62%), accuracy (99.37%), and specificity (99.66%) for 17 different types of arrhythmias using ECG data collected during the study. The fusion of deep learning models with gradient boosting optimizers represents the cutting edge of ensemble approaches for arrhythmia detection in 2024–2026, enabling sophisticated multi-lead analysis and feature integration. Rahman et al.^27^ represented a 12-lead electrocardiogram automatic classification model based on model fusion (CBi-DF-XGBoost) utilized XGBoost to fuse 12 single-lead CNN-BiLSTM models and domain-specific features, extracting local features through convolutional neural networks and temporal features through bi-directional long short-term memory, with fivefold cross-validation demonstrating macro-average F1 scores increasing by 12.0% and 14.0% for detecting paroxysmal arrhythmias compared with ResNet and LSTM respectively. Tenepalli et al.^28^ proposed an optimization techniques have significantly enhanced ensemble performance, with an optimized ensemble model developed using combination of 2D CNN for automatic feature extraction and LSTM for temporal prediction employing grid search optimization to tune hyperparameters, which provides the best hyperparameters despite computational expense, achieving improved arrhythmia classification performance while avoiding overfitting. Clinical value of advanced meta-learners has been shown when using Gradient Boosting, CatBoost, LightGBM, SVM and Neural Networks within a Hybrid Ensemble Learning framework with XGBoost as the meta-learner to predict cardiovascular risk and achieve an AUC-ROC of 0.82, Guan et al.^29^ while SMOTE was applied to mitigate the effect of imbalanced datasets and Interpretability methods such as SHAP, PCA, and tSNE were used to interpret the model. Chen et al.^30^ presents hybrids of Long Short-Term Memory (LSTM) and XGBoost architectures are the focus of previous work that have produced effective computational solutions for developing a classification system for predicting health risks related to patients with suspicious chest pain or angina. In this respect, multiple models were compared and evaluated in respect to performance regarding support vector machine (SVM), K-nearest neighbor (KNN), decision tree, XGBoost, random forest (RF), generalized boosted decision tree (GBDT), CatBoost, and Light Gradient Boosting Machine (LightGBM), with the results showing that XGBoost and LSTM provided the best overall predictions. The use of ensemble learning methods, specifically RF and GBDT including, but not limited to, XGBoost, have also been effective in making predictions about heart disease due to their capability to manage issues with missing patient data, regularization ability, and scalability. Advanced classification models, such as Benmalek et al. presented XGBoost and LightGBM, perform at a higher-level than traditional logistic regression and decision tree classifiers. The preceding developments have prompted subsequent work on feature-driven and hybrid learning techniques in arrhythmia and bundle branch block (BBB) classification. In one approach, Kaya et al.^31^ propose the use of higher order statistical and temporal features followed by a feature selection stage including forward search, backward elimination and genetic algorithm in order to get a very high accuracy model, although this handcrafted features only based model cannot well handle complexity of ECG. Al-Naami et al.^32^ combined the decomposition capabilities of MODWT-based wavelet with statistical features to learn the underlying representation in ECG and feed to ANFIS classifier that result in better performance than most of previous models. The limitation of this technique is dependence on preprocessing and high computational complexity. Hu et al.^33^ proposes a deep multi-instance learning framework for BBB classification without beat-level annotations overcoming labeling issues, but limited interpretability due to its complex architecture, and its result depends on aggregation mechanism of different instances. Dong et al.^34^ developed a transformer-based model with deformable attention, allowing better modeling of global features on multi-label ECG classification, but it requires massive dataset, huge computation and training data. In other work, Sun et al.^35^ coupled CNN with BiGRU with attention for better modeling temporal features. Sun et al.^36^ suggests an EEMD-denoised CNN-LSTM-SE network in order to capture more effective feature representation however they require lots of computational resources and sensitive to dataset. Similarly, Nazia et al.^37^ added the CBAM attention mechanism on the top of BiLSTM framework to improve classification accuracy; this architecture becomes more complex. Time–frequency domain-based approaches can exploit both spectral and temporal information; in a work, Prakash et al.^38^ combined DWT and HT-WVD along with CNN-LSTM architecture to obtain higher accuracy, however, they are still dependent on the handcrafted preprocessing methods and lacks of end-to-end optimization of the whole system. Combination of CNN and Transformer has also shown its potential in modeling the local–global dependency of ECG signal, in the work of Alghieth et al.^39^ an improved CNN-Transformer framework is proposed but it leads to high computation complexity. In order to achieve interpretability in ECG classification, Kolliyil et al.^40^ proposes a framework based on clinically relevant features extracted from 12-lead ECG which leads to better explanation on the prediction but limits the expressive power of the model itself. Alyahya et al.^41^ proposes a computationally efficient, lightweight hybrid model and claims that it achieves better results than previous lightweight architectures and is well suited for devices with resource constraints. Gracy et al.^42^ proposes a residual dilated temporal transformer based on the edge deployment requirements. Advanced hybrid model architectures like Xiong et al.^43^ have also emerged. Gated fusion mechanism is proposed between CNN and Transformer branches. Explainable deep learning-based ECG analysis systems are investigated by Talukder et al.^1^, enhancing the trustworthiness through the use of XAI techniques. Sathi et al.^44^ focuses on the usage of federated learning to address privacy concerns and it relies on the handcrafted features extraction methods. A recent approach is presented by Kim et al.^45^, which proposes to forgo R-peak detection by using Stockwell Transform as a representation, this reduces the burden and enhances robustness but the computation is still increased. Albarrak et al.^46^ developed an optimized CNN-LSTM model using Gray Wolf Optimizer, and it shows convergence improvement while still relying on the preprocessing pipeline.

As evidenced by these studies, there is a convergence of methods for the use of ensemble methodologies, optimization methods, and hybrid architectures for the development of clinically deployable systems for the detection of arrhythmias that exhibit high performance across populations of patients and different recording conditions. Though there have been major advances recently in using automated ECGs to detect heart arrhythmias many of the major challenges have not been solved. (a) Single modeling architecture cannot represent electricity morphologically, in the temporal rhythm it develops through time, or in way that captures long term and historically related events. (b) Time–frequency representations (e.g., CWT scalograms) are typically used as static preprocessing artifacts rather than adaptive feature spaces. (c) Most ensemble methods employ static fusion rules, ignoring model-specific reliability and validation performance. (d) High-accuracy models often rely on synthetic data augmentation or exhibit excessive computational complexity, limiting clinical deployment. (e) Poor generalization, class imbalance, high computational complexity, and limited multi-scale feature integration remain inadequately addressed in existing ECG classification methods. Tables 1 and 2 presents a previous study, highlighting human-centric applications. Table 1 focuses on MIT-BIH ECG arrhythmia classification, incorporating recent human-based research, while Table 2 extends the comparison to diverse physiological signals and Table 3 shows performance comparison between existing approaches and the proposed system for diverse diseases, signal types, and image-based data.

**Table 1:** Summary of studies using ECG in MIT-BIH arrhythmia classification.

Former studies	Database	Methods/techniques	Limitations	Acc (%)	F1 (%)
Benmalek et al (2022)^51^	MIT-BIH, BIDMC	CWT + Micro-CNNs	Costly convolutions, Patient noise, computational cost and privacy	-	99.5
Król-Józaga et al (2022)^52^	MIT-BIH AFIB	CNN + Spectrogram, Scalogram, and Attractor Reconstruction	Preprocessing needs	94	94
Mohonta et al (2022)^53^	MIT-BIH arrhythmia	CWT + 2DCNN	Data quality, annotation reliance, Fourier errors, temporal loss	99.65	-
Senturk et al (2023)^54^	MIT-BIH, BIDMC	AlexNet + CWT	High loss rates, limited mixed-class studies, weak preprocessing, poor accessibility	98.67	-
Scarpiniti et al (2024)^55^	MIT-BIH arrhythmia	CWT + 2D CNN	Fusion gaps, noisy feature extraction, no novel architecture	98.48	98.46
He et al (2025)^56^	MIT-BIH Arrhythmia	BiLSTM, Multi-head attention Mechanism	Dataset imbalance caused many [Q] beats to be misclassified as [N], lowering precision	94.41	70.02
Kaya et al (2020)^31^	MIT-BIH arrhythmia	KNN, SVM, NN	Relies on handcrafted features with limited robustness and generalization	99.82	-
Al Naami et al. (2022)^32^	MIT-BIH Arrhythmia	MODWT	Limited scalability and generalization due to reliance on handcrafted features and ANFIS	99.88	99.90
Hu et al (2020)^33^	MIT-BIH arrhythmia, CPSC	CNN	Limited interpretability and generalization	78.58	-
Dong et al (2023)^34^	CPSC	CNN + ViT	High computational cost and limited generalization	-	82.9
Sun et al (2023)^35^	MIT-BIH arrhythmia, CPSC	CNN + attention-based RNN	Computationally intensive with limited long-range dependency modeling and generalization	-	91.10
Sun et al (2024)^36^	MIT-BIH arrhythmia	CNN + LSTM + SE	Computationally intensive with limited global dependency modeling and generalization	98.5	98
Najia et al (2025)^37^	MIT-BIH arrhythmia	CNN + CBAM + BiLSTM	High computational complexity with limited generalization and scalability across datasets	99.20	98.29
Prakash et. al (2026)^38^	MIT-BIH arrhythmia	Time–frequency (DWT, HT-WVD) + CNN-LSTM	High complexity + limited generalization	99.6	98.98
Alghieth et al (2025)^39^	MIT-BIH arrhythmia, PhysioNet 2017 Challenge	DeepECG-Net	High computational cost with limited real-time scalability and generalization	98.2	97.5
Kolliyil et al (2025)^40^	PTB XL	12-lead ECG + interpretable feature extraction + ML classifiers	Handcrafted features limit complexity and generalization	-	79%
Alyahya et al (2026)^41^	MIT-BIH arrhythmia	Parallel Hybrid Model (PHM)	Limited scalability and generalization under resource constraints	99	98
Gracy et al (2026)^42^	MIT-BIH arrhythmia	Hybrid 1D Conv + transformer	Reduced capacity limits generalization and performance on complex data	99.34	99
Xiong et al (2025)^43^	MIT-BIH arrhythmia	Hybrid CNN-Transformer Network with Gated Fusion	High complexity with limited generalization	99.46	97.11
Talukder et al (2025)^1^	MITDB, PTBDB and NSTDB	CNN/BiLSTM + attention/XAI	Increased complexity with limited efficiency and generalization	99.98	99.43
Sathi et al (2025)^44^	MIT-BIH arrhythmia	Federated learning + ECG fiducial feature extraction + explainable classification	Communication cost and handcrafted features limit scalability and generalization	92	91
Kim et al (2025)^45^	MIT-BIH arrhythmia	S-transform + CNN + Transformer (no R-peak detection)	High complexity with limited scalability	99.58	-
Albarrak et al (2025)^46^	MIT-BIH arrhythmia	1D + CNN + LSTM	Depends on segmentation and has limited generalization	97	-

**Table 2:** Summary of studies using different physiological signals.

Former studies	Database	Methods	Limitations	Acc (%)	F1 (%)
Jayalakshmy et al. (2020)^47^	RALE, Think labs Lung sound library	CWT + EMD + CNN models	Resource-heavy, patient variability	83.78	-
Mashrur et al (2021)^57^	Apnea-ECG database v1.0.0	SCNN + CWT	Slow manual screening, high cost of PSG, device accuracy issues	81.86	69.63
Narin et al (2022)^49^	Bern-Barcelona Database	pre-trained CNN models	Limited data, channel similarity, manual features, shallow improvement	92.27	92.21
Kaur et al (2022)^58^	Max Hospital, Saket	CWT + 15-layer CNN	Inter-ictal activity remains underexplored with limited studies and dataset coverage	91.7	92.3
Iqbal et al (2023)^59^	CAD	Google Net CNN, DL-PPG	Low-quality PPG signals, bias, small sample	83.8	-
Kumar et al (2023)^50^	CAP SDRC	INSOMNet system	Limited participants and small datasets, lack of scalability, high model com-plexity, performance issues, Computational intensity	98.68	-
Negi et al (2025)^60^	BCI Competition data IV 2b	CWT + MViT	Limited data availability, signal noise and variability, low accuracy and risk of over-fitting, need for stronger data augmentation, lack of generalization	72.89	72.82

**Table 3:** A comprehensive analysis of the performance of prior studies across heterogeneous diseases, signal and image modalities.

Authors	Methods/model	Accuracy (%)
Alshahrani et al. (2024)^61^	Dense-Net121-MobileNet-RF hybrid model	97.7
Asiri et al. (2025)^62^	Geometric active contour segmentation with fused CNN features for cancer classification	99.8
Shamsan et al. (2023)^63^	CNN-based feature extraction + feature fusion + classification	99.1
Alshahrani et al. (2023)^64^	Multi-CNN feature fusion for DR classification	99.7
Ahmed et al. (2023)^65^	Fusion of multi-CNN features for improved multi-class skin lesion classification	98.4
Kumar et al. (2023)^66^	Gaussian-optimized dense network for cardiovascular prediction	95
Kumar et al. (2023)^67^	Hawks optimizer	97
Arunachalam et al. (2022)^68^	Optimized ML for cardiovascular prediction	96
Saranya et al. (2025)^69^	DenseNet‑ABiLSTM	87.7
Kaya et al. (2021)^31^	KNN, SVM, NN	99.8

## Proposed ScaHybNet framework pipeline

### Data acquisition

The ECG Heartbeat Categorization Dataset (MIT-BIH Arrhythmia) analyzed in the current study is available in the Kaggle repository^20,21^, which is the most widely used reference dataset for the development, training, and testing of methods for ECG-based arrhythmia categorization and identification. In total, there are 48 half-hour, dual-channel ambulatory ECG recordings from 47 patients with variable ages and genders and a wide spectrum of clinically significant arrhythmias, like ventricular, supraventricular, and junctional arrhythmias, etc. There are 5 classes of heartbeat, which correspond to five major super classes of output labels (N, S, V, F, Q). Additionally, there are expert R-peak times and rhythm labels available, which could stand as ground truth labels for training and testing. Because of their standardized form, expert annotation, and assured MIT-BIH Arrhythmia Database, PhysioNet database^21^ availability, MIT BIH records came to be recognized as the gold standard reference dataset for benchmarking effort in the development and comparison of different methods for ECG signal-based cardiac arrhythmia identification and categorization.

### Flow chart

**Fig. 4:**
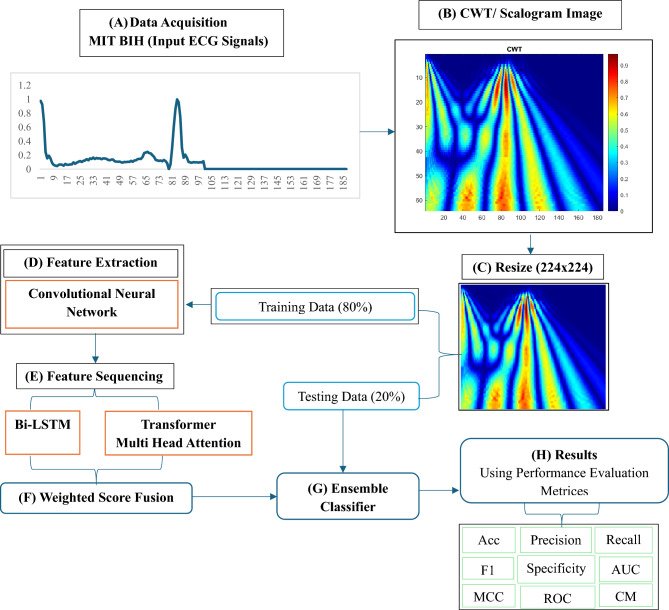
Flowchart diagram representing the proposed framework.

The entire work flow chart of the ScaHybNet framework is presented visually in Fig. 4 showing all the sequential processes that exist between processing an ECG signal, generating its scalogram with Continuous Wavelet Transform (CWT), performing hybrid feature learning and performing an ensemble classification.

### Methodology

**Fig. 5:**
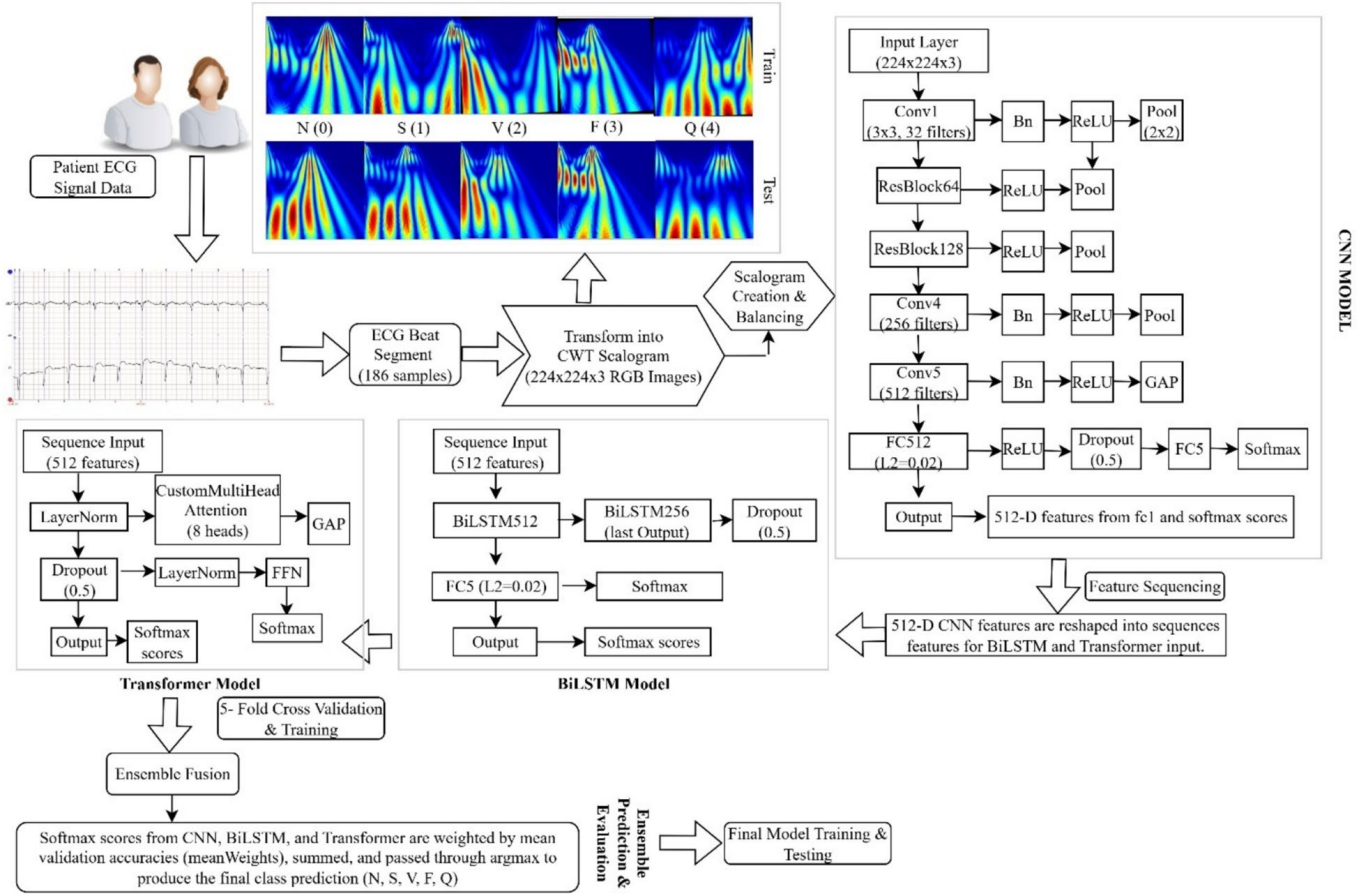
Block diagram representing the proposed methodology.

ScaHybNet consists of a triadic architecture that consists of complementary spatial, contextual, and temporal representations achieved through parallel learning so that the result can be used for robust arrhythmia classification. The block diagram (Fig. 5) illustrates how the semantic-level temporal modeling via feature-to-sequence reprojection can be accomplished without using the raw ECG sequence as input directly to the model. Figures 4 and 5 jointly describe the proposed ScaHybNet framework, where Fig. 4 summarizes the overall processing flow and Fig. 5 provides a detailed architectural view of the scalogram hybrid ensemble network model (ScaHybNet).

#### Data preprocessing

The utilized ECG Heartbeat Categorization Dataset consists of ECG signals that are already preprocessed and segmented, with each segment corresponding to an individual heartbeat. Beyond the above baseline preprocessing, further preprocessing stages are included in the proposed method to support strong representation learning of different heartbeat classes. Those stages involve class balancing, Continuous Wavelet Transform (CWT) based scalogram extraction using the Morlet wavelet, and deep feature pre-processing. These stages are designed to convert ECG signals into strong, learnable representations by retaining their physiological meaning and limiting human intervention. The ECG heartbeat MIT-BIH dataset involves ECG signals from human subjects, and the training and test datasets are split into two CSV files. In each one of the samples, there are 187 attributes, where the first 186 are for the ECG signal sample, and the last attribute is for the class label of each heartbeat sample. Each row stands for an ECG beat, and every beat is represented as a constant-length vector of 186 time-domain ECG samples. The ECG beats are pre-segmented and normalized into a fixed length that would allow machine learning analysis. Heartbeat classes are given by the numbers N = 0, S = 1, V = 2, F = 3, and Q = 4. Signals are unit-normalized to allow comparison among different patients’ ECG readings. The imbalanced distribution of ECG datasets is one of the main issues that makes detecting rare classes difficult, and performing with high accuracy is another challenge. Therefore, in this work, we use multiple approaches for both the data level and the model level. Firstly, class balancing is done in the preprocessing stage, and the number of samples in each class is adjusted to equal the same threshold during the training process. Secondly, the class weights with inverse frequency are applied during the training to penalize misclassifying minority classes. Furthermore, an adaptive weighted ensemble is used to provide more robust results by adaptively combining the predictions of the different branches. The complete ECG data preprocessing pipeline is formally described in Algorithm 1.

**Algorithm 1:**
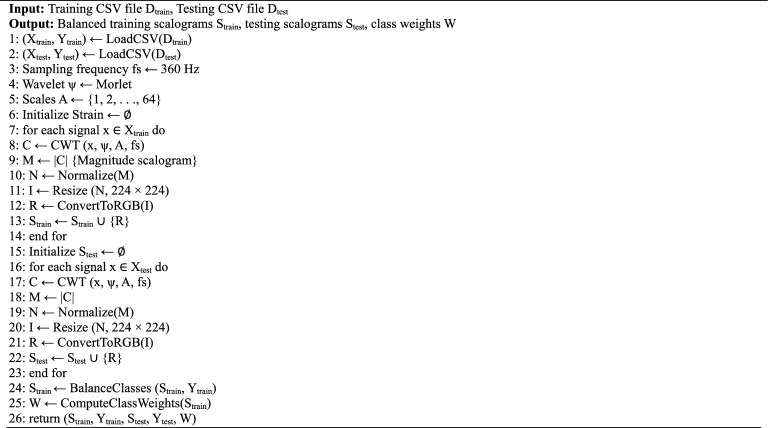
ECG data preprocessing.

The MIT-BIH Arrhythmia ECG recordings are available in the ECG Heartbeat Categorization Dataset on Kaggle^20^ as CSV Files, with one row per ECG segment and the last column containing the arrhythmia class label for each segment. The process of pre-processing has removed the signals and arrhythmia class labels from the original signal so that they can be transformed independently and used for supervised learning. To maintain temporal consistency across samples and to avoid variations in scale due to time–frequency analysis, all ECG signals undergo processing at a fixed 360 Hz sampling frequency that meets MIT-BIH guidelines. The process of transforming one-dimensional electrocardiographic signals to two-dimensional time–frequency representations involves using continuous wavelet transforms (CWT) that utilize Morlet wavelets to achieve such transformations. Specifically, ECG signals were transformed into time–frequency scalogram representations using the Continuous Wavelet Transform defined as Eq. 1:

1 $$W(a,b)=\frac{1}{\sqrt{a}}{\int}_{-\infty }^{\infty }x(t){\psi }^{*}\left(\frac{t-b}{a}\right)dt$$

where *a* is the scale parameter, *b* denotes translation, *x(t)* is the ECG signal, and *ψ* represents the mother wavelet. The CWT parameters used in this study were: Sampling frequency: 360 Hz, Mother wavelet: Morlet (‘morl’), Scale range: 1–64, Scalogram image size: 224 × 224 RGB images generated for deep feature learning. The Morlet wavelet was selected because of its well-established suitability for non-stationary biomedical signals such as ECGs. In particular, its Gaussian-windowed sinusoidal structure provides:

- Excellent joint time–frequency localization, enabling simultaneous preservation of transient and spectral information;
- Effective characterization of ECG morphological variations, particularly QRS complexes, P- and T-wave components, and subtle ST-T abnormalities;
- Multi-resolution analysis capability, allowing low-frequency rhythm characteristics and high-frequency abrupt changes to be captured across different scales;
- Superior suitability for scalogram generation, producing informative two-dimensional texture patterns that enhance convolutional feature extraction.

A multiscale analysis was implemented on all transformed signals. In detail, a fixed scale range (1 to 64) was used to evaluate signals, and then the model was enabled to learn rhythm information at low frequency and morphology patterns (like QRS complexes and ST-T variation) at high frequency. Here, a range from 1 to 64 scales was picked to obtain a wide variety of information in an ECG trace (from local finer patterns up to rhythm information), and we resized the resulting scalograms to 224 × 224 to suit the proposed deep architecture. Comparison with db4 Wavelet: Moreover, we compared Morlet with the Daubechies-4 (db4) wavelet, which is very popular for ECG denoising and QRS detection (Table 4).

**Table 4:** Comparative analysis of wavelets.

Wavelet	Characteristics	Suitability for study
Morlet	Continuous, oscillatory, strong time–frequency localization	Highly suitable for scalogram generation and deep feature learning
db4	Compactly supported, discrete wavelet, excellent for QRS detection	Strong for signal decomposition but less effective for rich continuous time–frequency imaging

**Fig. 6:**
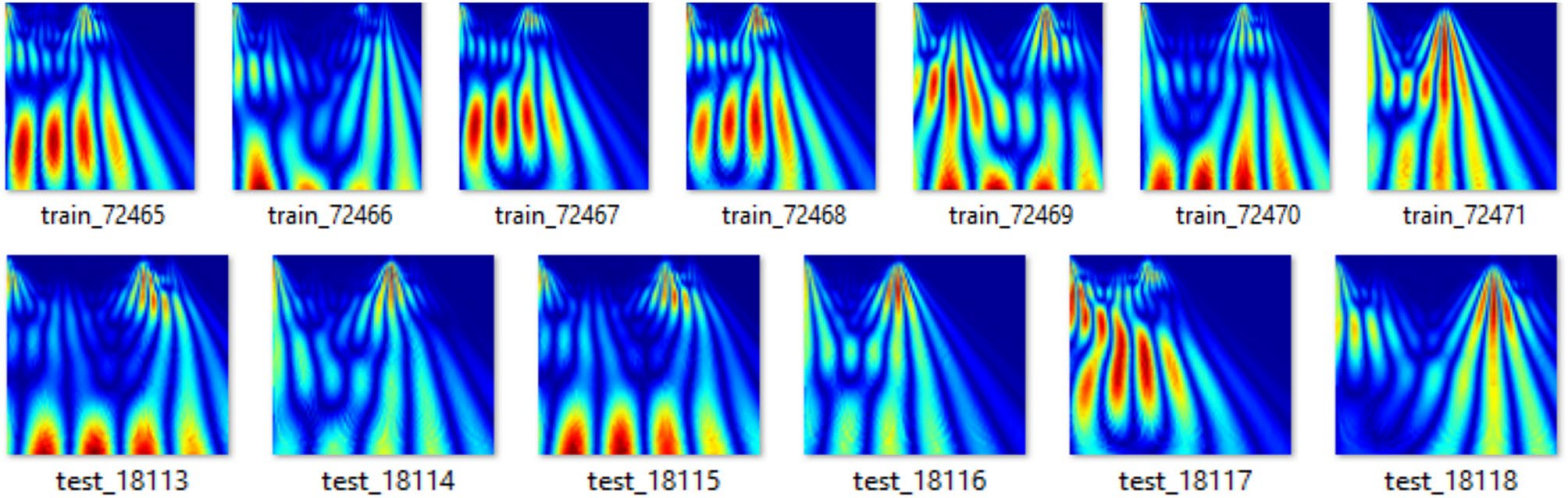
Scalogram (**a**) Train samples (**b**) Test samples.

While db4 is highly effective for discrete wavelet decomposition and beat delineation, it is primarily optimized for signal decomposition rather than continuous scalogram image generation. In contrast, Morlet produces smoother and more informative continuous time–frequency representations, which are particularly advantageous for image-based deep learning frameworks such as the proposed ScaHybNet. Preliminary comparative experiments further indicated that Morlet-generated scalograms yielded superior discriminative texture patterns and improved classification performance relative to db4-based representations. Accordingly, Morlet was adopted as the preferred mother wavelet in the proposed framework. Further to create scalograms of the CWT’s magnitude of coefficients, scalograms can be used to display the energy profile of a signal over both time and frequency. To reduce amplitude fluctuations between samples when using a scalogram to aid in learning stability, the scalograms are normalized so that they fall within a fixed intensity range. The normalized scalograms have been resized to a consistent spatial size of 224 × 224 pixels related to the standard operating procedures of deep CNN architectures. Consequently, the grayscale scalograms were converted into a color image form (RGB space) using one of the various available colormaps to take advantage of features learned from previous image classification tasks on vision models. Scalogram images are stored in a certain directory structure that is specific to the class they belong to so the folder names provide a way to automatically infer the label for each image. An image datastore is created for both training and testing, with a way to quickly load batches of images and shuffle them while the model is being trained. Figure 6 presents representative scalogram samples generated from the MIT-BIH dataset using the proposed preprocessing pipeline, as stored in the class-wise directories (i.e., N, S, V, F, Q) created during training.

##### Model training and classification (ScaHybNet architecture and ensemble design)

Unique Contributions of ScaHybNet: The proposed ScaHybNet differs from existing hybrid ECG classifiers through several key innovations that collectively contribute to its novelty and improved discriminative capability.

1. Residual Scalogram Feature Learning: ScaHybNet employs a custom residual convolutional neural network (CNN) with skip connections to extract discriminative spatial features from two-dimensional ECG scalograms. The residual learning mechanism is represented as Eq. (2).
  2 *y=F(x,W)+x*
  where $F\left(x,W\right)$ denotes the learned nonlinear transformation and *x* is the identity mapping. This residual formulation preserves fine morphological structures in ECG scalograms, mitigates degradation in deeper networks, and enhances multilevel spatial feature representation compared with conventional CNN feature extractors.
2. Hierarchical Temporal Modeling via Stacked BiLSTM: To capture temporal dynamics embedded in the extracted feature representations, ScaHybNet incorporates two stacked bidirectional long short-term memory (BiLSTM) layers. The bidirectional hidden representation can be expressed as Eq. (3).
  3 $${h}_{t}=[\overrightarrow{{h}_{t}};\overleftarrow{{h}_{t}}]$$
  where forward and backward hidden states jointly model both short-term beat dynamics and long-range rhythm dependencies. This hierarchical temporal modeling provides complementary sequential information beyond spatial feature extraction alone.
3. Custom Multi-Head Self-Attention Feature Interaction Module (Role of the custom attention mechanism):The most specific distinguishing innovation in ScaHybNet is the design of a novel multi-head self-attention feature interaction module to learn global context relations between the extracted features, which is different from existing CNN-BiLSTM models that mostly depend on the memories in recurrent networks. Equation (4) explains how the attention module interacts with features dynamically by weighted queries, keys, and values (4):
  4 $$Q=X{W}_{Q},K=X{W}_{K},V=X{W}_{V}$$
  with attention computed as equation (5).
  5 $$Attention(Q,K,V)=softmax\left(\frac{Q{K}^{T}}{\sqrt{d}}\right)V$$

This mechanism enables adaptive emphasis on informative feature relationships rather than treating all dependencies uniformly. To further enhance contextual representation, the module employs eight parallel attention heads, defined as equation (6).

6 $$hea{d}_{i}=Attention({Q}_{i},{K}_{i},{V}_{i})$$

And combined through at Eq. (7).

7 $$MultiHead=Concat(hea{d}_{1},\dots ,hea{d}_{8}){W}_{O}$$

Through this multi-head formulation, multiple heads can learn to focus on different relationships, such as local morphological interrelations, long-range contextual interdependence, finer arrhythmic interdependence, and multi-scale discriminative dependencies, respectively, which makes the attention mechanism more powerful than typical recurrent modules can offer with their representation ability. Moreover, the attention module adds rather than overlaps with the role of Bi-LSTM; the Bi-LSTM considers the temporal dependency along the time step, whereas the attention module deals with global feature dependency. They jointly provide modeling of dependency through temporal memory and contextual attention, which are normally absent in typical two-branch hybrid models. Table 5 shows the role of a custom multi-head self-attention module.

**Table 5:** Attention specific comparative analysis highlighting the role of the proposed custom multi-head self-attention module in ScaHybNet.

Attention-specific capability	Existing hybrids	ScaHybNet
Standard sequential dependency modeling only	✓	No
Custom query-key-value projections	Rare	✓
Eight-head parallel dependency learning	Rare	✓
Attention-guided feature refinement	Rare	✓
Global + temporal dual dependency modeling	Rare	✓
Adaptive fusion of attention outputs	Rare	✓

**Fig. 7:**
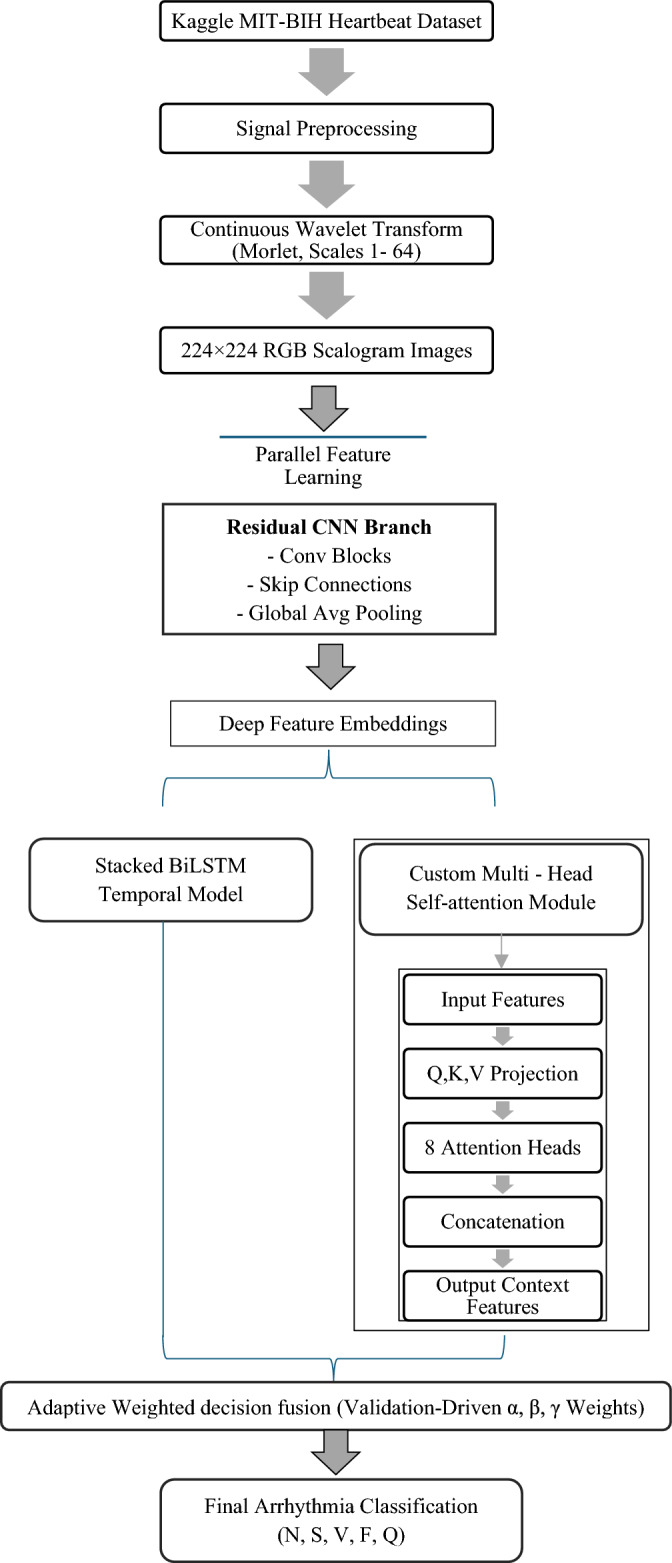
Architecture of the proposed ScaHybNet framework with custom multi-head self-attention and adaptive weighted ensemble fusion.

(4) Adaptive Weighted Ensemble Fusion:A further novelty of ScaHybNet lies in its adaptive weighted ensemble fusion strategy. Instead of simple averaging or static fusion, outputs from the CNN, BiLSTM, and attention branches are combined through validation-driven weighted fusion:
  8 $$S=\alpha {S}_{CNN}+\beta {S}_{BiLSTM}+\gamma {S}_{Attn}$$
  where *α*, *β*, and *γ* denote adaptive branch weights determined according to predictive reliability. This dynamic decision-level fusion enables each branch to contribute proportionally to its discriminative strength and enhances robustness under class imbalance, constituting another major contribution of the proposed framework. As illustrated in Fig. 7, the proposed ScaHybNet framework integrates custom multi-head self-attention with an adaptive weighted ensemble fusion strategy, allowing effective aggregation of spatial, temporal, and global contextual features for enhanced ECG classification performance. Overall, the novelty is we introduced custom multi-head attention as a global dependency modeling branch and fused it adaptively with CNN and BiLSTM.

**Fig. 8:**
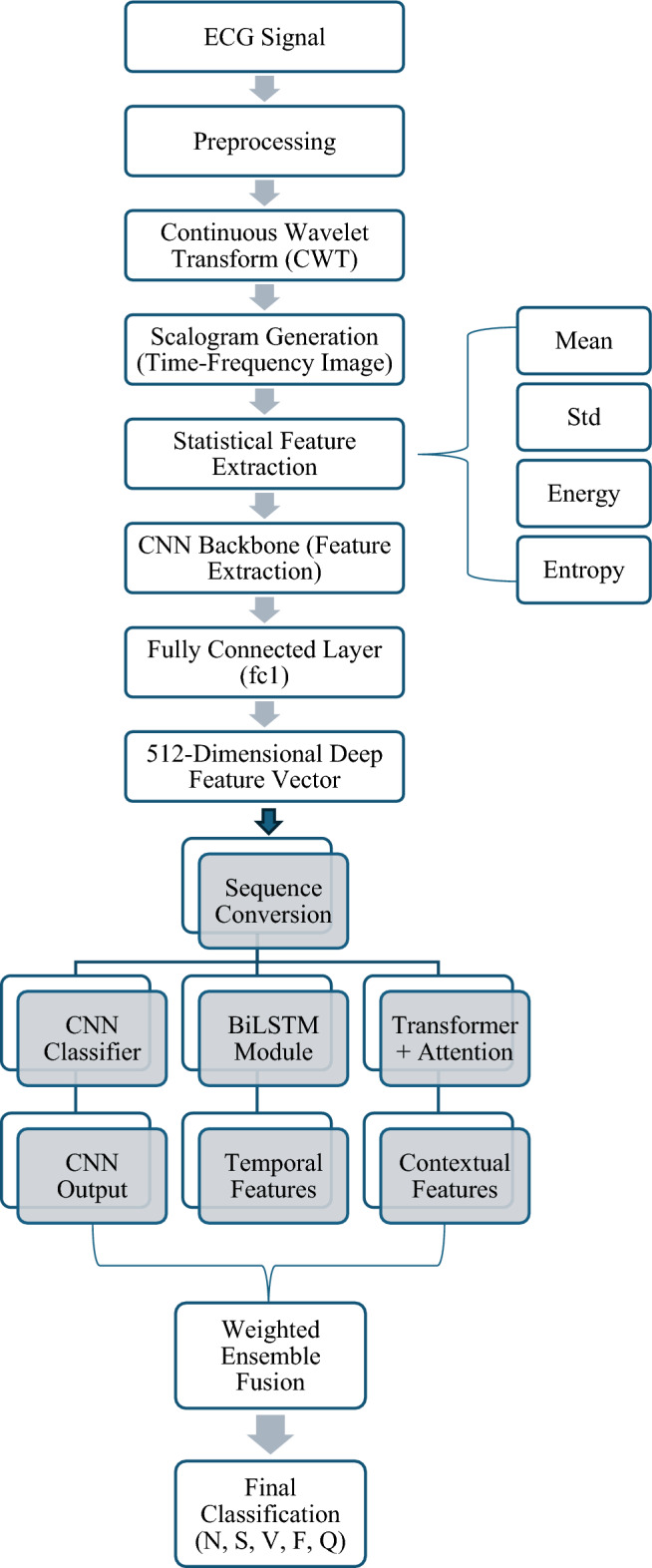
Data feature extraction process.

**Fig. 9:**
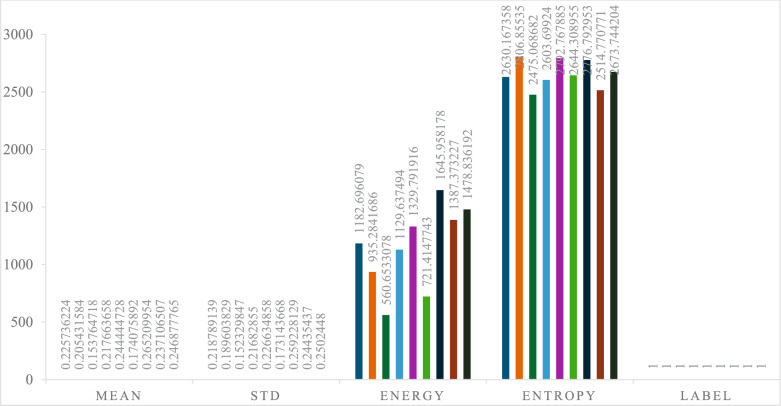
Statistical features extraction.

**Fig. 10:**
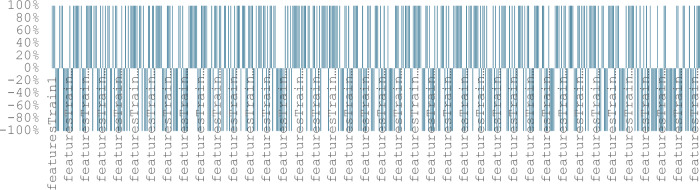
Deep features extraction (512 features).

Figure 8 presents a diagram of data feature extraction process. In the proposed ScaHybNet framework, statistical features are explicitly extracted from the scalogram representations obtained via continuous wavelet transform (CWT). These features, including mean, standard deviation, energy, and entropy, capture the distribution and complexity of ECG signal patterns. Figure 9 presents a few samples signals statical features bar graph. In parallel, the scalograms are processed by a CNN to learn high-level deep features, where the fully connected layer (fc1) produces a 512-dimensional feature vector, extracted deep features shows at Fig. 10. Both statistical and deep features are integrated to create a holistic feature representation that is then employed by BiLSTM and Transformer-based attention modules for temporal or contextual modeling to enhance classification accuracy and robustness. Statistical and deep features include robust spatial and morphological patterns of the ECG signal. Preprocessing and feature extraction efficiency would appear as better class separation and smooth model convergence during the training process of the model, and prove that the proposed pipeline can contribute to robust classification for the ECG signals. In the results section, Table 20 compares current hybrid ECG classifiers with the ScaHybNet framework with respect to architecture, self-attention, and weighted fusion method.

The architectural parameters of the CNN, BiLSTM, and attention modules were determined. Training hyperparameters, including learning rate, batch size, number of epochs, and regularization factors, to ensure stable convergence and optimal performance while avoiding overfitting. The below hyperparameters were selected at Table 6 based on empirical tuning using five-fold cross-validation to achieve optimal performance while maintaining computational efficiency.

**Table 6:** Detailed hyperparameter settings and configuration of the proposed ScaHybNet architecture.

Component	Parameter	Value/setting	Description/justification
Input	Input size	224 × 224 × 3	Scalogram image representation
CNN	Filter sizes	32, 64, 128, 256, 512	Hierarchical feature extraction
	Kernel size	3 × 3	Standard for spatial feature learning
	Residual connections	Yes	Mitigates vanishing gradient
	Pooling	Max pooling (2 × 2)	Dimensionality reduction
	Fully connected layer	512 neurons	Feature embedding
	Dropout	0.5	Reduces overfitting
	L2 regularization	0.02	Prevents overfitting
BiLSTM	Layers	2	Temporal hierarchy modeling
	Hidden units	512, 256	Short- and long-term dependency capture
	Output mode	Last	Sequence summarization
	Dropout	0.5	Regularization
Attention module	Number of heads	8	Multi-scale contextual learning
	Projection dimension	512	Feature space consistency
Training	Optimizer	Adam	Fast and stable convergence
	Learning rate	1 × 10^−4^	Empirically optimized
	Batch size	2	Memory-constrained training
	Epochs	150	Ensures convergence
	Learning rate schedule	Piecewise (drop factor 0.1)	Improves convergence stability
Regularization	Dropout (Global)	0.5	Prevents overfitting
	Class weights	Inverse frequency-based	Handles class imbalance
Validation	Cross-validation	5-Fold	Robust performance estimation
Data processing	Wavelet type	Morlet (morl)	Good time–frequency localization
	Scales	1–64	Multi-resolution representation

**Fig. 11:**
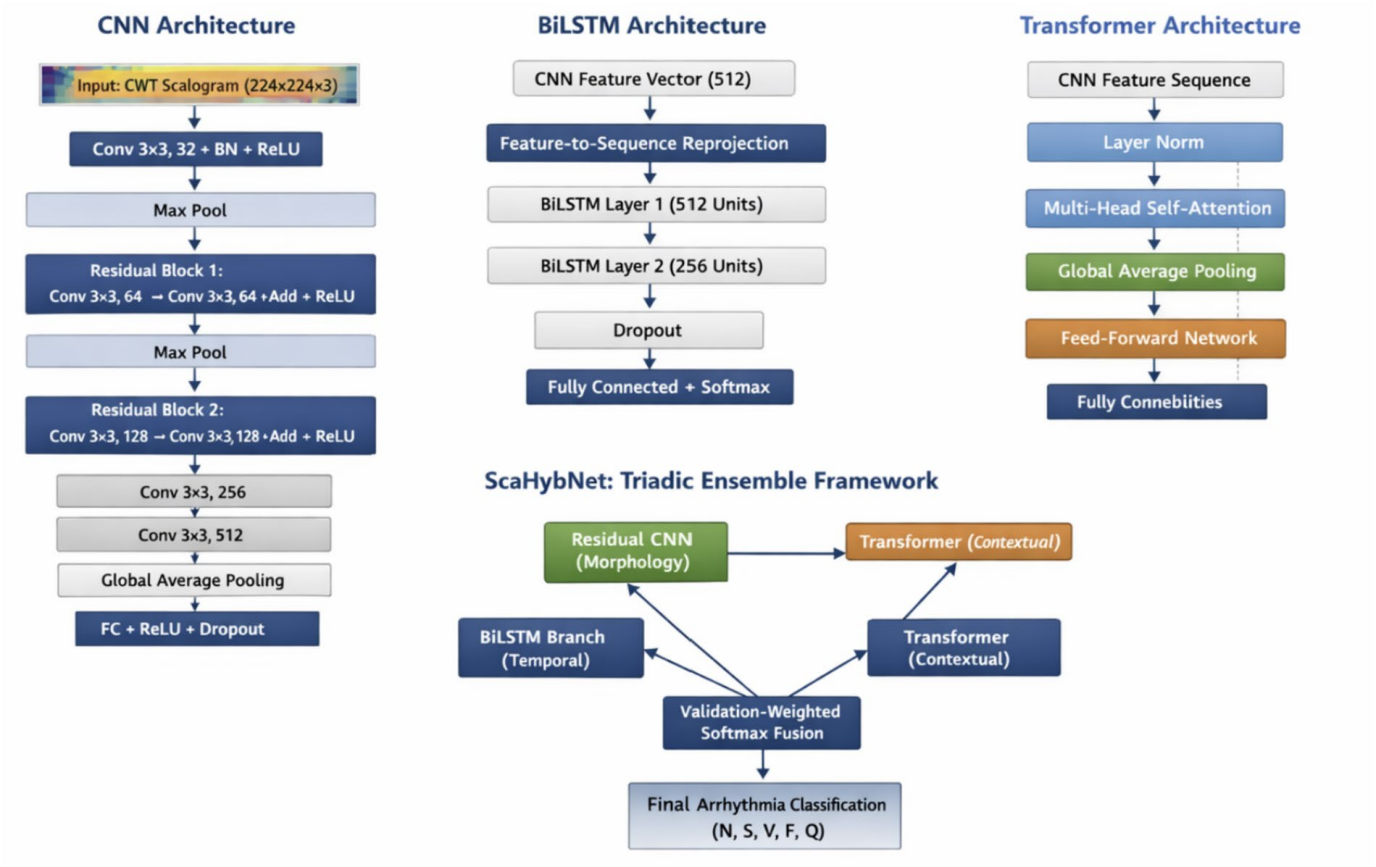
Architecture of the proposed ScaHybNet framework for ECG arrhythmia classification, illustrating (**a**) the residual CNN-based morphology encoder operating on CWT scalograms, (**b**) the BiLSTM branch with feature-to-sequence reprojection for temporal modeling, (**c**) the Transformer branch for long-range contextual dependency learning using multi-head self-attention, and (**d**) the triadic ensemble structure with validation-weighted softmax fusion for final arrhythmia classification.

Figure 11 shows a system architecture of the ScaHybNet Framework for Arrhythmia Classification, which ensures a fast and powerful classification system. The framework will initially extract each patient’s distinct morphological characteristics on the CWT-based scalogram representation using a CNN. After extracting these features, they will be fed into two parallel branches: the first is BiLSTM branch for temporal modeling of heart rhythm sequence; the second is the Transformer branch for learning long-distance context of the heart rhythm sequence. An average, weighted by their performance on the validation set, will be performed over all predictions to combine these two branches’ contributions toward final diagnosis. Algorithm 2 demonstrates the entire training and classification procedure of the proposed framework, and Algorithm 3 shows an alternative performance-aware ensemble fusion mechanism to the above fixed averaging strategy. Algorithm 4 explains a mechanism to reprojection features to sequence, which makes the framework more semantic by not merely capturing the recurrence of the raw signal but the context modeling through treating the CNN features as a structured sequence.

**Algorithm 2:**
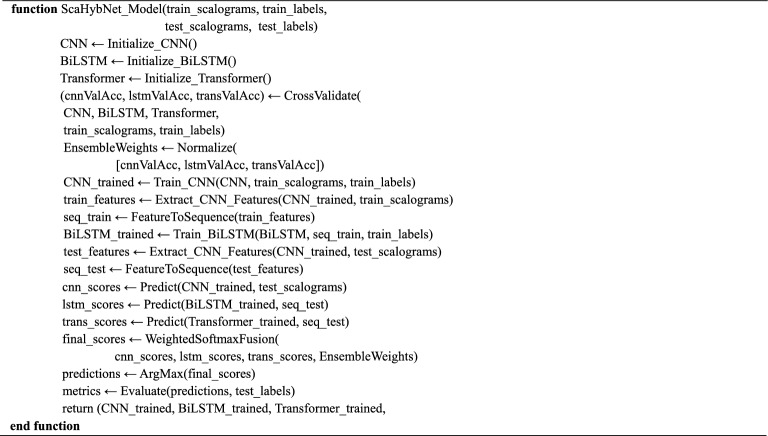
ScaHybNet based ECG arrhythmia classification.

**Algorithm 3:**
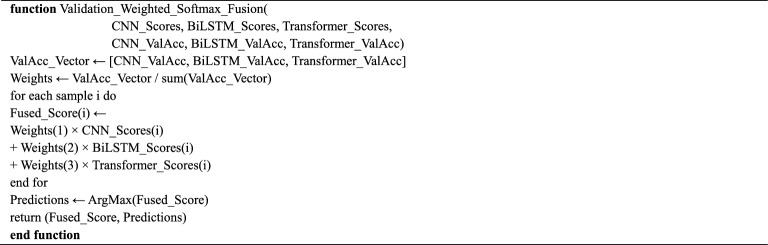
ScaHybNet based ECG arrhythmia classification.

**Algorithm 4:**
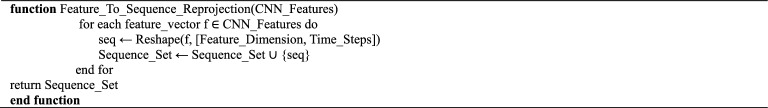
ScaHybNet based ECG arrhythmia classification.

After generating balanced CWT-based scalogram representations, the proposed ScaHybNet framework performs arrhythmia classification through a structured multi-stage learning pipeline: (a) CNN-Based Spatial Feature Extraction—Input images of CWT scalograms are forwarded to the residual CNN to extract low-level morphological and spatial features representing different aspects of ECG waveforms. The residual connection can maintain detailed diagnostic information and facilitate better gradient flow during training. (b) Feature Embedding Generation—Global average pooling and a fully connected layer can process the CNN-produced spatial feature maps into a 512-dimensional feature vector of individual ECG signals to learn morphological characteristics. (c) Temporal Feature Modeling via BiLSTM—The CNN embeddings are reshaped as sequences and are fed into a BiLSTM for bidirectional modeling of the temporal dependence and progression of rhythm on ECG. (d) Global Context Modeling using Transformers-The same CNN embeddings are separately fed to a multi-head self-attention transformer model to model the long-term dependency without explicitly relying on R-peak detection. (e) Cross-Validation-Based Model Training—CNN, BiLSTM, and Transformer models are trained in a cross-validation scheme of 5-folds, and their validation accuracy can be recorded. (f) Validation-Weighted Ensemble Fusion—Predictions from CNN, BiLSTM, and Transformer are integrated by softmax fusion, where weights are assigned to models according to their validation accuracy. (g) Final Training and Evaluation (Inference and Metrics)—Retrained the ensemble model on the entire balanced training set and tested it on the unseen test set to perform the final evaluation using metrics including accuracy, precision, recall, F1-score, specificity, MCC, ROC curve, and AUC.

##### Mathematical notation

The mathematical notation described in Eq. (9)-(43) fully outlines each phase of the proposed framework. In the same order, it covers signal translation by CWT, feature extraction, time modeling, and attentive learning. The notation provides an analytical and systematic representation of the multi-scale spatial–temporal modeling and adaptive fusion.

1. Signal representation and time frequency transformation
  Let an ECG heartbeat signal be:
  9 $$x\in {\mathbb{R}}^{T},T\approx 187$$

The Continuous Wavelet Transform (CWT) is defined as:

10 $$C(a,b)=\frac{1}{\sqrt{a}}{\int}_{-\infty }^{\infty }x(t)\hspace{0.17em}{\psi }^{*}\left(\frac{t-b}{a}\right)\hspace{0.17em}dt$$

where,

- $$a\in {\mathbb{R}}^{+}$$: scale
- $$b\in {\mathbb{R}}$$: translation
- *ψ*: Morlet wavelet

Discretized scalogram:

11 $$S=\mid C(a,b)\mid \in {\mathbb{R}}^{M\times N}$$

After normalization and resizing:

12 $${S}^{^{\prime}}=\mathcal{R}(\mathcal{N}(S))\in {\mathbb{R}}^{224\times 224\times 3}$$

2. Residual CNN Feature Extraction

Let:

13 $$S^{\prime}F^{\left( l \right)} {\text{(feature map at layer }}l)$$

Convolutional transformation:

14 $$F^{\left( l \right)} = \sigma \left( {W^{\left( l \right)} *F^{{\left( {l1} \right)}} + b^{\left( l \right)} } \right)$$

Residual connection (as implemented in your skip layers):

15 $$F^{\left( l \right)} = {\mathcal{H}}\left( {F^{{\left( {l1} \right)}} } \right) + F^{{\left( {l1} \right)}}$$

where:

16 $$\mathcal{H}(F)=\sigma ({W}_{2}*\sigma ({W}_{1}*F))$$

Global pooling:

17 $$g=\frac{1}{HW}\sum_{i=1}^{H}\sum_{j=1}^{W}{F}_{i,j}^{\left(L\right)}$$

Final embedding:

18 $$z=\phi ({W}_{fc}g+{b}_{fc})\in {\mathbb{R}}^{512}$$

3. Sequence Construction

So, each sample becomes:

19 $$z\in {\mathbb{R}}^{512\times 1}$$

Thus sequence:

20 $$Z=\{{z}_{1},{z}_{2},\dots ,{z}_{n}\}$$

4. Bi-LSTM temporal modeling

Forward pass:

21 $$\overrightarrow{{i}_{t}}=\sigma ({W}_{i}{z}_{t}+{U}_{i}{h}_{t-1}+{b}_{i})$$

22 $$\overrightarrow{{f}_{t}}=\sigma ({W}_{f}{z}_{t}+{U}_{f}{h}_{t-1}+{b}_{f})$$

23 $$\overrightarrow{{o}_{t}}=\sigma ({W}_{o}{z}_{t}+{U}_{o}{h}_{t-1}+{b}_{o})$$

24 $$\overrightarrow{{\widetilde{c}}_{t}}=\mathrm{t}\mathrm{a}\mathrm{n}\mathrm{h}({W}_{c}{z}_{t}+{U}_{c}{h}_{t-1}+{b}_{c})$$

25 $$\overrightarrow{{c}_{t}}={f}_{t}\odot {c}_{t-1}+{i}_{t}\odot {\widetilde{c}}_{t}$$

26 $$\overrightarrow{{h}_{t}}={o}_{t}\odot \mathrm{t}\mathrm{a}\mathrm{n}\mathrm{h}({c}_{t})$$

Backward pass similarly:

27 $$\overleftarrow{{h}_{t}}$$

Final BiLSTM representation:

28 $${h}_{t}=[\overrightarrow{{h}_{t}};\overleftarrow{{h}_{t}}]$$

5. Custom Multi-Head Self-Attention (Deep Formulation)

Mathematically:

29 $$Q=X{W}_{Q},K=X{W}_{K},V=X{W}_{V}$$

Scaled dot-product:

30 $$A=\frac{Q{K}^{T}}{\sqrt{{d}_{k}}}$$

Softmax normalization:

31 $${\alpha}_{ij}=\frac{\mathrm{e}\mathrm{x}\mathrm{p}({A}_{ij})}{\sum_{j}\mathrm{e}\mathrm{x}\mathrm{p}({A}_{ij})}$$

Attention output:

32 *Z=α V*

Multi-Head Structure

33 $${head}_{i}=Attention(X{W}_{Q}^{i},X{W}_{K}^{i},X{W}_{V}^{i})$$

34 $$H=Concat({head}_{1},\dots ,{head}_{8})$$

35 $${Z}_{attn}=H{W}_{O}$$

Interpretation.

Each head learns:

- local morphological dependencies
- long-range temporal correlations
- class-specific discriminative patterns

6. Branch-wise Prediction

Each branch outputs logits:

36 $${S}_{CNN}=softmax({W}_{c}z)$$

37 $${S}_{BiLSTM}=softmax({W}_{b}h)$$

38 $${S}_{Attn}=softmax({W}_{a}{Z}_{attn})$$

7. Adaptive Weighted Fusion

Weights from validation:

39 $$\alpha =\frac{Ac{c}_{CNN}}{\sum Acc}\beta =\frac{Ac{c}_{BiLSTM}}{\sum Acc}\gamma =\frac{Ac{c}_{Attn}}{\sum Acc}$$

Final prediction:

40 $$S=\alpha {S}_{CNN}+\beta {S}_{BiLSTM}+\gamma {S}_{Attn}$$

8. Loss Function

Cross-entropy:

41 $$\mathcal{L}=-\sum_{i=1}^{C}{y}_{i}\mathrm{l}\mathrm{o}\mathrm{g}({\widehat{y}}_{i})$$

With class weights:

42 $$\mathcal{L}=-\sum {w}_{i}{y}_{i}\mathrm{l}\mathrm{o}\mathrm{g}({\widehat{y}}_{i})$$

9. Final Prediction
  43 $$\widehat{y}=\mathrm{a}\mathrm{r}\mathrm{g}\mathrm{m}\mathrm{a}\mathrm{x}S$$

## Experimental performance metrics results, discussion & interpretability analysis

### Hardware setup, training configuration and hyperparameters

This methodology focuses on quantifying data related to the human ECG signal while ensuring reproducibility of results through ethical practices and clinically relevant methods for data handling. The calculations necessary to complete this process occurred in MATLAB R2024a using an NVIDIA T1000 GPU (4 GB of VRAM). A set of hyperparameters were used during the training of the model, including the use of an Adam optimizer with an initial learning rate equal to 1e-4, using a piecewise learning rate schedule that dropped every 10 epochs by 0.1; using mini-batch sizes between (2—128) and allowing the model to run for up to 200 maximum epochs, while shuffling the data every epoch, and validating once every 10 iterations. The validation patience was set equal to infinity, allowing for the model to run until fully trained without early stopping, and allows for an in-depth examination of the degree of overfitting on human data. All computations were completed on a GPU-enabled compute environment to maximize the efficiency of calculations and used verbose output along with training progress plots to show the performance of the model. Recent research into the application of continuous wavelet transforms (CWT) to human ECG data has provided additional supporting detail regarding the use of the Morlet wavelet as the most preferred form for detecting transient events, as well as providing additional improved accuracy through the use of scalogram fusion techniques.

### Evaluation metrics & experiment results

**Fig. 12:**
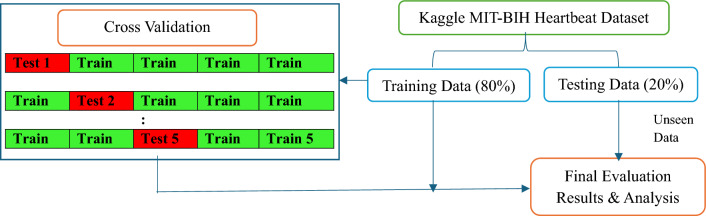
Dataset split strategy for model evaluation.

**Fig. 13:**
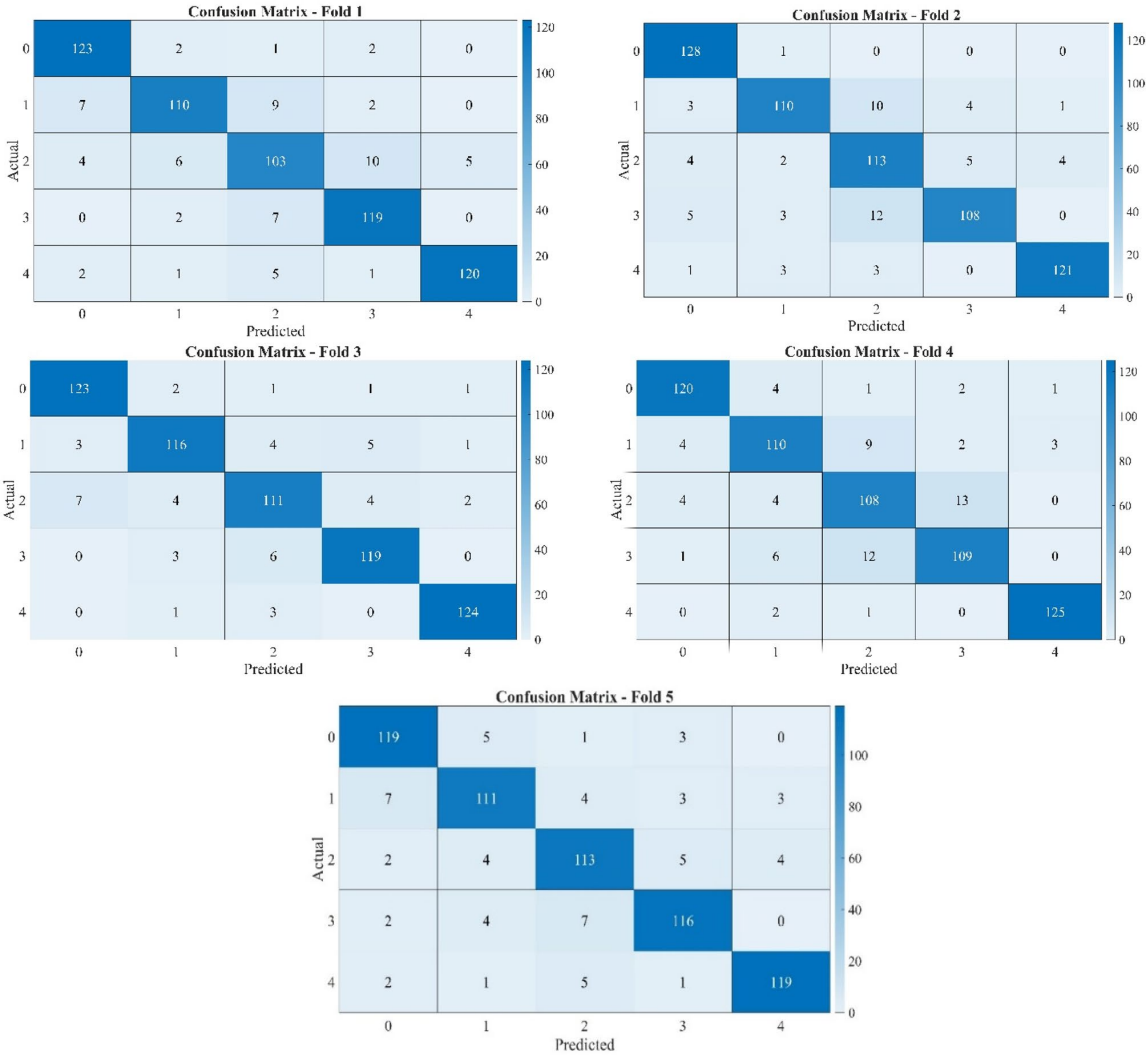
5- fold CV confusion matrix.

**Fig. 14:**
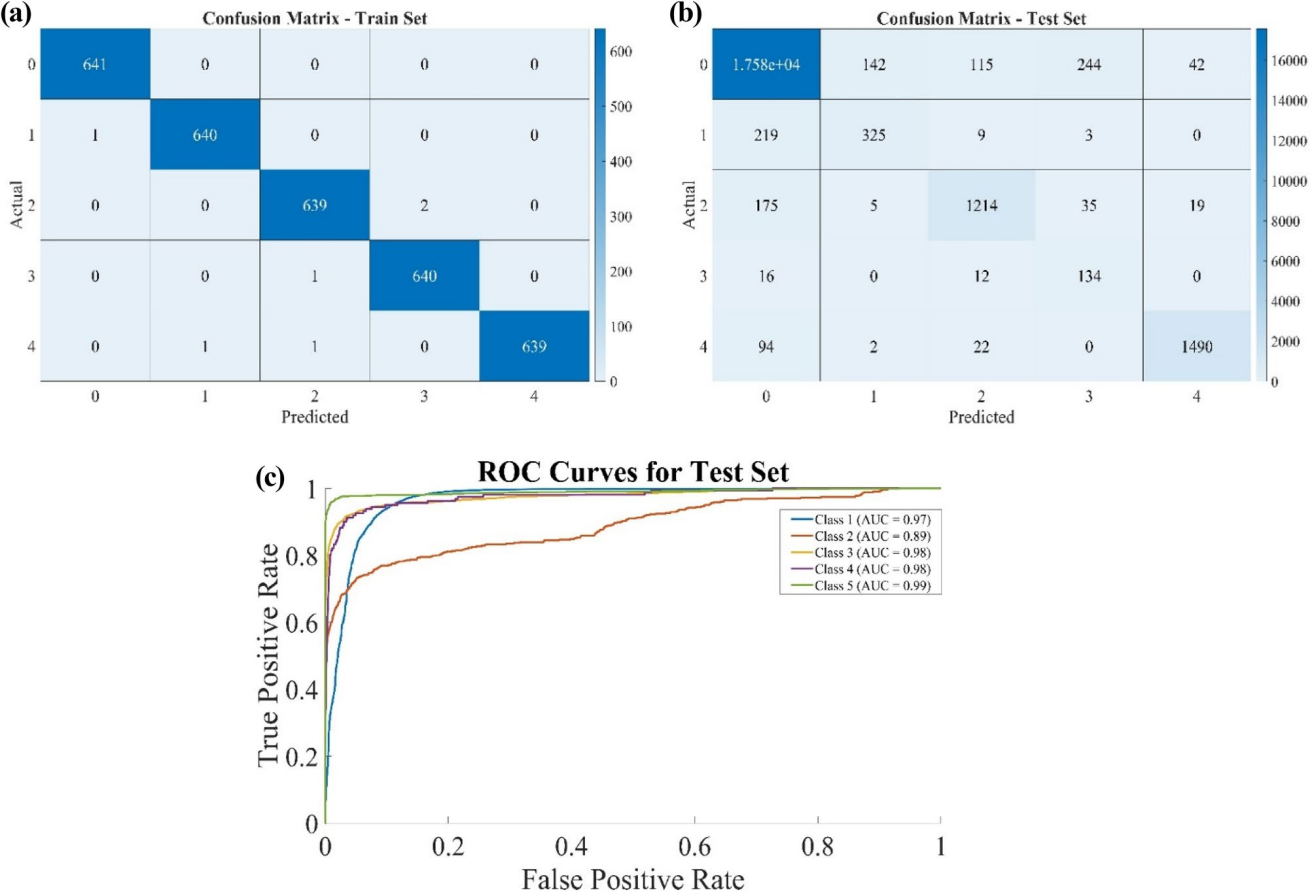
(**a**) Confusion matrix for train set (**b**) confusion matrix for test set (**c**) Roc curve for test set.

The results from ScaHybNet model confirmed the validity of our experiments through fivefold cross-validation (mean accuracy of 90.42% ± 1.26%). At optimal hyperparameters (batch size = 2; number of epochs = 150), ScaHybNet reached an ensemble training accuracy of 99.81% and an ensemble test performance of 94.73% accuracy, with 76.51% precision, 82.93% recall, and a macro F1 score of 77.40%. Our hyperparameter sweeps revealed that a batch size of 2 was the optimal setting, as using larger batch sizes produced overfitting due to gradient instability. Additionally, as we increased the number of epochs beyond 150, there was a diminishing return on the improvement in accuracy (92.28% at 200 epochs). Both the minority classes had difficulties being classified, particularly Supraventricular (F1 = 63.12%) and Fusion (F1 = 46.37%). However, the weighting of the minority classes helped to some extent, but F1 scores of ~ 50–60% indicated they were still negatively impacted by suboptimal hyperparameter settings. Figure 20 the Batch Size Accuracy Chart illustrates test accuracies spanning 91.99% (batch 128) to 94.73% (batch 2), while Fig. 21 the Generalization Gap Chart highlights gaps ranging from 3.83% (batch 128) to 7.76% (batch 64), outperforming the literature average of 7.9%. Table 7 shows a fold-wise and mean performance of fivefold cross-validation on training data. Table 8 reports the per-class test metrics for batch size 2 over 150 epochs. Figure 12 shows the train-cross-validation test split strategy, while Fig. 13 presents the five-fold cross-validation confusion matrices. Figure 14 further provides the confusion matrices for both the training and test sets, along with the ROC curve for the test set. Table 9 compares performance measures for the different batch sizes at 150 epochs while Table 10 compares the weighted F1 scores on the test set over all five classes at 150 epochs. Table 11 compares the different measures of performance over the different epoch numbers while batch size is held at 64.

**Table 7:** Fold-wise and mean performance of fivefold cross-validation on training data.

Epoch	Batch size	Fold 1	Fold 2	Fold 3	Fold 4	Fold 5	Mean 5- CV fold accuracy ± Std deviation
150	2	89.70	90.48	92.51	89.24	90.17	90.42% ± 1.26%

**Table 8:** Per-class test metrics (batch sizes 2, 150 epochs) on MIT BIH dataset.

Class	Precision (%)	Recall (%)	F1 Score (%)	Specificity (%)	MCC (%)
N (0)	97.21	97.00	97.12	86.65	83.31
S (1)	68.56	58.45	63.12	99.30	62.43
V (2)	88.48	83.84	86.09	99.23	85.18
F (3)	32.21	82.72	46.37	98.70	51.11
Q (4)	96.07	92.66	94.33	99.70	93.91
Macro average	76.51	82.93	77.40	96.72	75.19

**Fig. 15:**
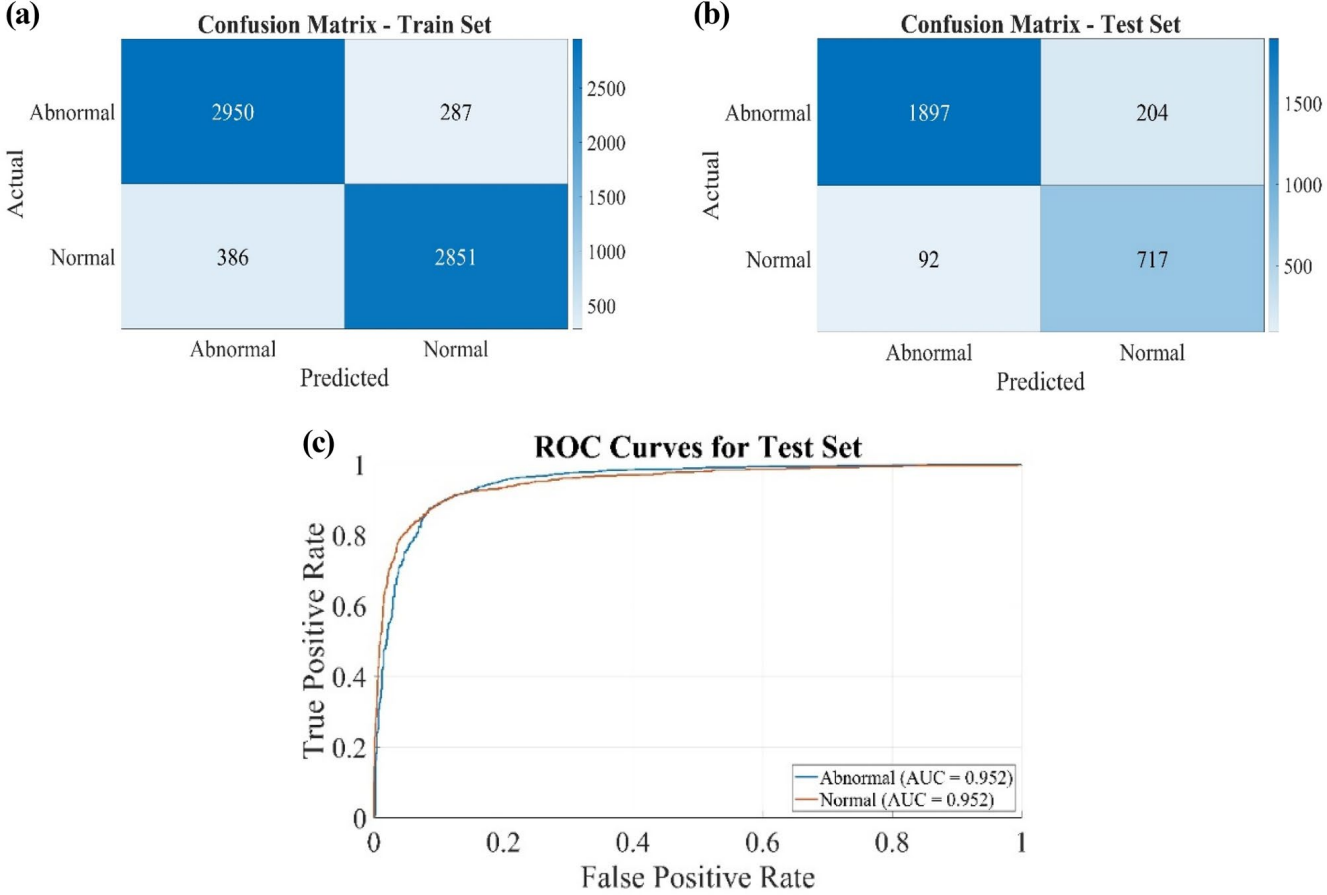
(**a**) Confusion matrix for train set (**b**) confusion matrix for test set (**c**) Roc curve for test set.

**Fig. 16:**
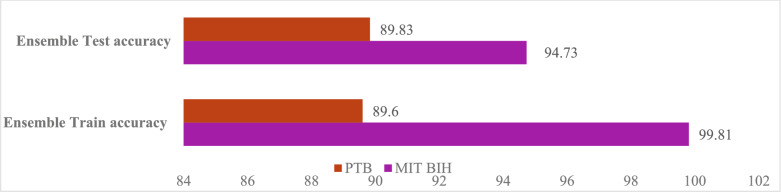
Comparison bar plots across both dataset.

**Table 9:** Performance metrics across batch sizes at 150 epochs on MIT BIH dataset. Bold significance is for the proposed model.

Epoch	Batch size	Set	Ens. accuracy (%)	Mean precision (%)	Mean recall (%)	Mean F1-score (%)
150	2	Train	99.81	99.81	99.81	99.81
		Test	**94.73**	**76.51**	**82.93**	77.40
150	4	Train	99.84	99.84	99.84	99.84
		Test	94.37	75.31	82.13	**77.70**
150	8	Train	**1**	1	1	1
		Test	94.45	75.06	82.19	76.40
150	16	Train	1	1	1	1
		Test	93.82	73.76	**82.93**	75.32
150	32	Train	1	1	1	1
		Test	93.89	73.5	80.81	74.65
150	64	Train	99.69	99.69	99.69	99.69
		Test	91.93	67.25	81.03	70.79
150	128	Train	95.82	95.84	95.82	95.82
		Test	91.99	68.23	78.68	70.68

**Table 10:** Weighted F1-scores for test set across five classes (150 epochs) on MIT BIH dataset. Bold significance is for the proposed model.

Epoch	Batch size	Ensemble train accuracy (%)	Ensemble test accuracy (%)	Weighted F1-score for test set (five classes)				
				0(N)	1(S)	2(V)	3(F)	4(Q)
150	2	**99.81**	**94.73**	3.203	0.098292	0.25598	0.028639	0.28427
150	4	99.84	94.37	3.2153	0.09867	0.25697	0.028749	0.28536
150	8	1	94.45	3.1616	0.097022	0.25268	0.028269	0.2806
150	16	1	93.82	3.117	0.095653	0.24911	0.02787	0.27664
150	32	1	93.89	3.0891	0.094796	0.24688	0.027621	0.27416
150	64	99.69	91.93	2.9295	0.089899	0.23413	0.026194	0.26
150	128	95.82	91.99	2.9249	0.089759	0.23376	0.026153	0.25959

**Table 11:** Performance metrics across epochs at batch size 64 on MIT BIH dataset. Bold significance is for the proposed model.

Epoch	Batch size		Ens. accuracy (%)	Mean precision (%)	Mean recall (%)	Mean F1-score (%)
20	64	Train	98.91	98.91	98.91	98.91
		Test	**93.23**	**71.33**	80.12	**73.39**
50	64	Train	98.47	98.48	98.47	98.47
		Test	92.23	67.78	80.96	71.65
100	64	Train	99.44	99.44	99.44	99.44
		Test	92.14	68.27	80.41	70.93
150	64	Train	99.69	99.69	99.69	99.69
		Test	91.93	67.25	81.04	70.79
200	64	Train	**99.72**	**99.72**	**99.72**	**99.72**
		Test	**92.28**	**68.67**	**81.06**	**72.07**

An information leak was avoided by carefully cross-validating training; this fivefold cross-validation was performed on the training partition, and a hold-out test partition was used solely for unseen evaluation after model training. Class balancing and inverse-frequency weighting were performed only on the training partition. Since the Kaggle MIT-BIH heartbeat benchmark that was used is already segmented as a beat-level dataset and does not contain patient identifications, a guaranteed subject-independent patient-wise separation is not possible and is mentioned as a limitation.

Proposed ScaHybNet attained mean fivefold cross validation accuracy 90.42%, a small standard deviation 0.0126, means that the results obtained at the fivefold cross-validation have stable stability. And a narrow range 95% confidence interval [0.8932, 0.9153] means the model stability not resulted from luck. A statistical significance analysis was performed using fivefold cross-validation result. Proposed ScaHybNet has average fivefold cross validation accuracy of 90.42% and small std 0.0126, indicating robust consistency across folds and confidence interval also indicates robustness. Besides, the ensemble training accuracy of 99.81% and test accuracy of 94.73% both exhibit remarkable learning and generalization capability. In the meanwhile, from Table 12, Paired t-test indicates that the proposed model is statistically significantly better than the Transformer based model (p < 0.001), but not statistically significantly better than CNN and BiLSTM model (p > 0.05). From this, it appears that the proposed ensemble structure can improve the robustness and generalization capability, especially over attention-based models.

**Table 12:** Statistical significance (paired t-test).

Comparison	p-value	Interpretation
ScaHybNet vs CNN	0.78812	Not significant
ScaHybNet vs BiLSTM	0.24198	Not significant
ScaHybNet vs transformer	0.00033	Highly significant

To further assess the generalizability of the presented method the analysis of results has been performed on different ECG datasets The PTB Diagnostic ECG Database from Kaggle^20^ when possible and comparable metric performance assessed using various standard evaluation methods Results were plotted using confusion matrices, receiver operating characteristic (ROC) curves and the comparison plot. Figure 15 shows the confusion matrices on train and test sets respectively as well as the ROC curve for the test set. By visualizing the metrics as described earlier we provide better analysis of performance per class and allow for better visual analysis and understanding of the models performance on multiple datasets.

**Fig. 17:**
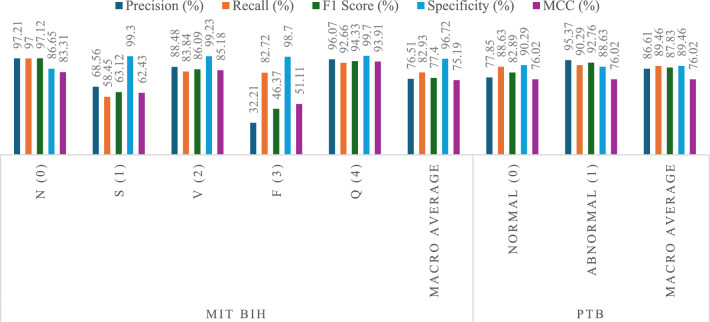
Per-class test metrics bar chart across both dataset.

In Fig. 16 are depicted the compare bar plot on both the data-sets. Figure 17 shows the per-class test metric bar-plot on both data-sets. Table 13 shows the per-class test metric on the PTB data-set. Table 14 reports the performance of proposed models on cross data-sets. In Table 15 the per-class test metric for two data-set have been reported and the comparison of per-class test metric for five classes after 150 epoch on two data-set are reported on Table 16.

**Fig. 18:**
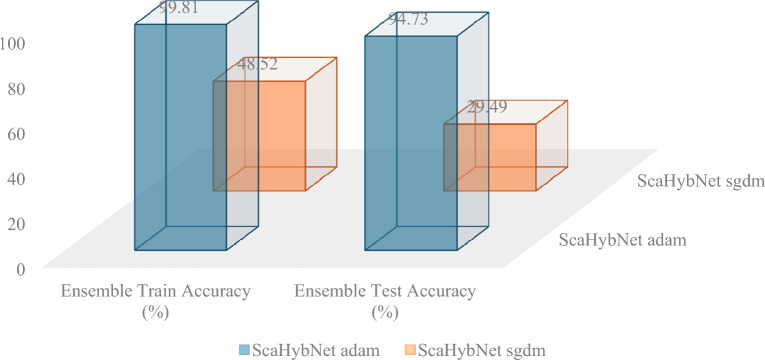
Comparative evaluation between different optimizers.

**Fig. 19:**
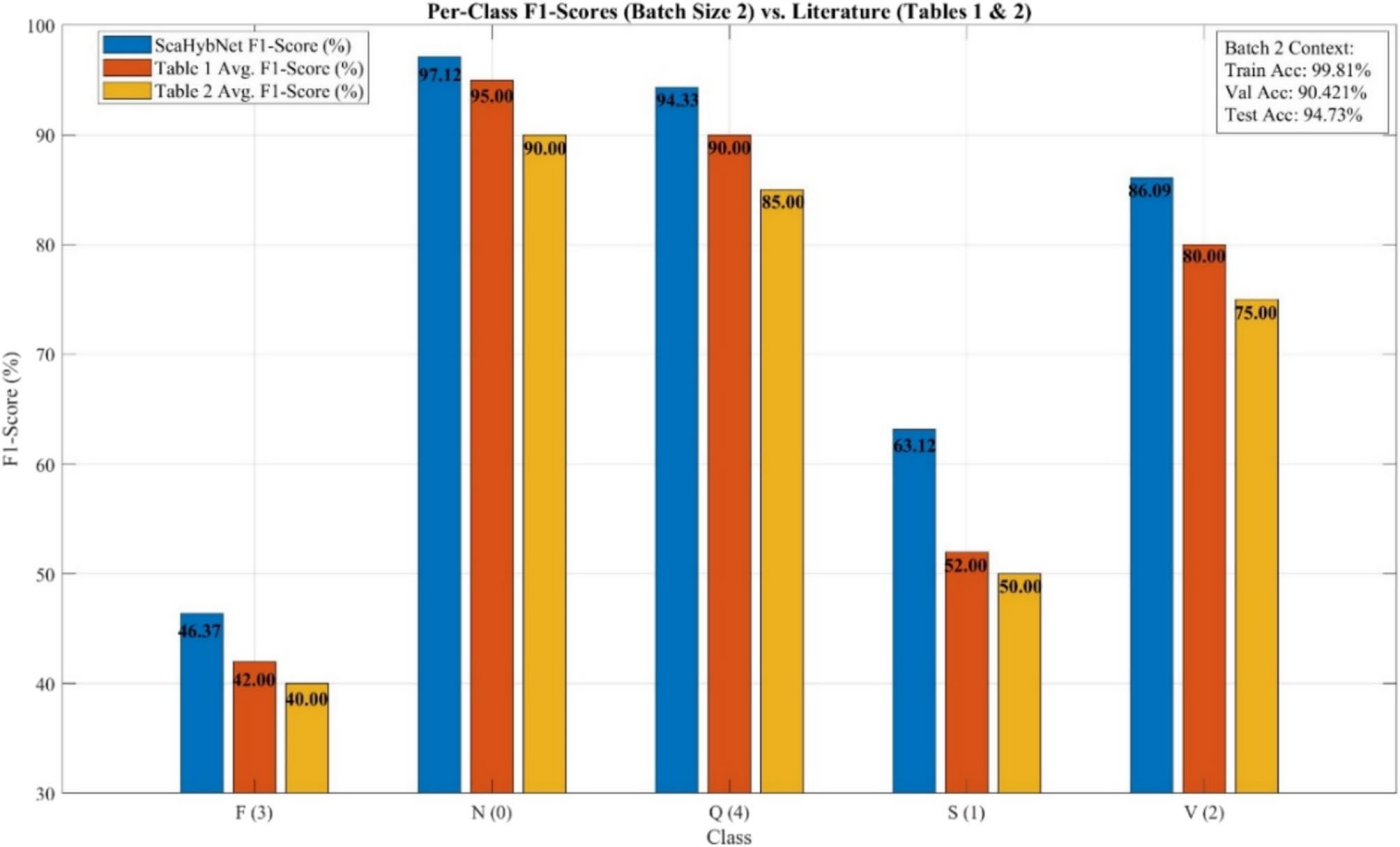
Comparative bar plot of class-wise F1-scores (%) achieved by ScaHybNet against state-of-the-art methods reported in the literature.

**Table 13:** Per-Class test metrics (batch size 2, 150 epochs) on PTB data.

Class	Precision (%)	Recall (%)	F1 score (%)	Specificity (%)	MCC (%)
Normal (0)	77.85	88.63	82.89	90.29	76.02
Abnormal (1)	95.37	90.29	92.76	88.63	76.02
Macro average	86.61	89.46	87.83	89.46	76.02

**Table 14:** Cross-dataset performance evaluation of the proposed ScaHybNet framework.

Cross datasets	Ensemble train accuracy	**Ensemble test accuracy**	5-fold CV mean accuracy:	Model size	Total training time (sec)
MIT BIH	99.81	94.73	90.42% ± 1.26%	28.90 MB	16,299,900
PTB	89.60	89.83	87.86% ± 1.62%	30.74 MB	3,259,980

**Table 15:** Comparative analysis per-class test metrics (batch size 2, 150 epochs) across both dataset.

Data	Class	Precision (%)	Recall (%)	F1 score (%)	Specificity (%)	MCC (%)
MIT BIH	N (0)	97.21	97.00	97.12	86.65	83.31
	S (1)	68.56	58.45	63.12	99.30	62.43
	V (2)	88.48	83.84	86.09	99.23	85.18
	F (3)	32.21	82.72	46.37	98.70	51.11
	Q (4)	96.07	92.66	94.33	99.70	93.91
	Macro average	76.51	82.93	77.40	96.72	75.19
PTB	Normal (0)	77.85	88.63	82.89	90.29	76.02
	Abnormal (1)	95.37	90.29	92.76	88.63	76.02
	Macro average	86.61	89.46	87.83	89.46	76.02

**Table 16:** Comparison table for weighted F1-scores for test set across five classes (150 epochs) for both dataset.

Dataset	Epoch	Batch size	Ensemble train accuracy (%)	Ensemble test accuracy (%)	Weighted F1-score for test set (five classes)				
					0(N)	1(S)	2(V)	3(F)	4(Q)
MIT BIH	150	2	99.81	94.73	3.203	0.098292	0.25598	0.028639	0.28427
					0 (N)			1(A)	
PTB	150	2	89.60	89.83	23.04			66.97	

In order to compare the performance of the feature extraction backbone proposed, various CNN architectures, such as LeNet 5 (which is lightweight) and deeper architectures that are better at extracting features, may be evaluated. Various trade-offs exist between these models in terms of complexity and performance. The CNN architecture utilized for this task is a custom residual CNN architecture, which is able to effectively extract hierarchical spatial features from the scalogram representation without incurring too much computational overhead. The residual connections improve feature propagation and help prevent the problem of vanishing gradients. Comparative insights with alternative CNN architectures provide a broader understanding of design trade-offs and further justify the selection of the proposed architecture within the ScaHybNet framework. Table 17 presents a comparative analysis of lightweight models along with their computational times. Table 18 compares the per-class test metrics obtained on the MIT-BIH dataset using Batch Size 2 and 150 epochs between the proposed ScaHybNet framework and the lightweight LeNet5 model.

**Table 17:** Comparative analysis of proposed framework with lightweight LeNet5 model.

Model	Model size (MB)	Training time (Sec)	Inference time (sec)	Total parameters	Ensemble train accuracy (%)	Mean fivefold CV accuracy (%)	Ensemble test accuracy (%)
ScaHybNet	28.90	16,299,900	312.85	8,850,629	99.81	90.42 ± 1.26	94.73
LeNet5	1.48	1565.30	54.96	389,439	58.28	56.25 ± 4.25	44.08

**Table 18:** Per-class test metrics (batch size 2, 150 epochs) on MIT BIH data comparison with lightweight LeNet5 model.

Model	Class	Precision (%)	Recall (%)	F1 score (%)	Specificity (%)	MCC (%)
ScaHybNet	N (0)	97.21	97.00	97.12	86.65	83.31
	S (1)	68.56	58.45	63.12	99.30	62.43
	V (2)	88.48	83.84	86.09	99.23	85.18
	F (3)	32.21	82.72	46.37	98.70	51.11
	Q (4)	96.07	92.66	94.33	99.70	93.91
	Macro average	76.51	82.93	77.40	96.72	75.19
LeNet5	N (0)	96.24	40.12	56.64	96.35	53.6
	S (1)	04.74	30.57	08.21	95.28	07.1
	V (2)	26.83	61.67	37.39	96.85	29.6
	F (3)	03.04	87.03	05.88	89.62	06.6
	Q (4)	42.15	73.13	53.48	95.92	47.6
	Macro average	34.60	58.51	32.32	94.80	28.9

While prior studies have reported strong performance across individual physiological domains, a component-level comparison reveals consistent architectural omissions. Table 19 shows the results from an ablation-based evaluation of several different methods across domains (biomedical fields) providing evidence of lack of spatial, temporal, contextual and fusion mechanisms that exist in these methods which justifies the unified framework of ScaHybNet as proposed here. Table 20 presents a comparative analysis between existing hybrid ECG classification models and the proposed ScaHybNet framework, highlighting architectural novelty, custom self-attention mechanisms, and adaptive weighted fusion strategies. Table 21 compares the overall performance of the proposed system with existing methods across different diseases and signal/image modalities.

**Table 19:** Comparative analysis of cross-domain methodological approaches across literature related to physiological signal processing.

Former studies	Physiological signal	Time–frequency representation	Spatial encoder	Temporal modeling	Attention/context modeling	Fusion strategy	Key Missing component (ablation perspective)
Jayalakshmy et al. (2020)^47^	Lung sounds	CWT + EMD	CNN	✗	✗	✗	No temporal or contextual modeling
Mashrur et al. (2021)^57^	Apnea ECG	CWT	Shallow CNN	✗	✗	✗	Temporal dependencies ignored
Narin et al. (2022)^49^	EEG	Pre-trained CNN	CNN	✗	✗	✗	No rhythm/context learning
Kaur et al. (2022)^58^	EEG	CWT	Deep CNN (15-layer)	✗	✗	✗	No sequence modeling
Iqbal et al. (2023)^59^	PPG	None/Raw	GoogLeNet	✗	✗	✗	No time–frequency or temporal modeling
Kumar et al. (2023)^50^	Multimodal (Sleep)	Handcrafted	Custom DL	Partial	✗	✗	Limited generalization, high complexity
Negi et al. (2025)^60^	EEG (BCI)	CWT	MViT	✗	✓	✗	No recurrent temporal modeling
**ScaHybNet (Proposed)**	ECG (MIT-BIH)	CWT Scalogram	Residual CNN	BiLSTM	Transformer	Validation-weighted fusion	–

**Table 20:** Comparative analysis of existing hybrid ECG classifiers and the proposed ScaHybNet highlighting architectural novelty, custom self-attention, and adaptive weighted fusion.

Authors	Hybrid architectures	Scalogram	Residual CNN	Recurrent modeling	Custom multi-head self-attention module	Adaptive weighted fusion	Three-branch spatial–temporal-attention integration
Kathayat et al (2025)^70^	Hybrid Deep Learning Model for Scalogram-Based ECG Classification	✓	No	Partial	No	No	No
Talukder et al (2025)^71^	Hybrid Multiscale Feature Fusion Model	No	Partial	Limited	No	No	No
Mohammed et al (2026)^72^	CNN-LSTM-GRU Hybrid	No	No	✓	No	No	No
Ye et al (2025)^73^	Hybrid CNN-BLSTM	No	No	✓	No	No	No
Tchinda et al (2025)^74^	ECG Shape-Adapted Wavelets + BiLSTM	Wavelet-Based	No	✓	No	No	No
Bakshi et al (2025)^75^	Cardiac Arrhythmia Detection Using 2-D ECG Scalograms	✓	Partial	Limited	No	No	No
Asole et al (2025)^76^	Multi-Class ECG Arrhythmia Detection Using Scalogram and DL	✓	Limited	No	No	No	No
Mhetre et al (2025)^77^	Hybrid Autoencoder-CNN	No	Partial	No	No	No	No
**Proposed**	ScaHybNet	✓	✓	✓	✓	✓	✓

#### Comprehensive ablation study analysis

We conducted a rigorous ablation analysis of ScaHybNet on the balanced MIT-BIH Arrhythmia dataset using five-fold cross-validation to systematically quantify the contribution of different design choices. Tables 8, 9, 10 and 11 show that the proposed framework achieved test accuracy of 94.73% and training accuracy of 99.81%, while the cross-validation accuracy was only 90.42%, although still competitive when looking at performance on minority classes (i.e., supraventricular [S] and fusion [F] beats) with F1-scores of 63.12% and 46.37%, respectively. Table 22 summarizes the results of removing either the Transformer branch or the BiLSTM branch, with reduced accuracies of 2.63% and 2.88%, respectively. Therefore, these findings support the importance of combining both temporal modeling and long-range contextual modeling in achieving high levels of accuracy. Additionally, replacing our proposed validation-weighted softmax fusion method with a uniform averaging approach resulted in a further loss of accuracy of 2.33%, indicating the importance of performance-aware methodologies for ensemble integration. The ablation results of feature level representation in Table 23 demonstrate that CWT scalogram representations provide an accuracy gain of 3.09% over STFT spectrograms, while also yielding significant improvements in minority class recall (fusion beats: 82.72% vs. 63.44%) and further validating the robustness of the cross-scale representation of time–frequency. In addition, Table 24 compares ScaHybNet to other previously published CWT based architectures (CWT based CNN’s, CNN-BiLSTM’s and CNN-Transformer’s) demonstrating that while the capacity to represent data is improved with each of these architectures, only with the full integration of the various components inherent to ScaHybNet (residual morphology encoding, semantic feature to sequence reprojection, dual temporal modeling and validation weighted fusion) is optimal and most consistent performance achieved. Ultimately, these multiple approaches to ablation analysis confirm ScaHybNet’s improvement is achieved through the synergistic nature of all the elements present within the architectural framework as opposed to just the individual model complexities indicating ScaHybNet remains well-positioned as an acceptable means of producing robust clinically relevant arrhythmia classification from ECG data.

**Table 21:** An overall performance evaluation where current systems are benchmarked against the proposed system in several diseases and signal/image modalities.

Authors	Methods/model	Results (accuracy %)
Alshahrani et al. (2024)^61^	Dense-Net121-MobileNet-RF hybrid model	97.7
Asiri et al. (2025)^62^	Geometric active contour segmentation with fused CNN features for cancer classification	99.8
Shamsan et al. (2023)^63^	CNN-based feature extraction + feature fusion + classification	99.1
Alshahrani et al. (2023)^64^	Multi-CNN feature fusion for DR classification	99.7
Ahmed et al. (2023)^65^	Fusion of multi-CNN features for improved multi-class skin lesion classification	98.4
Kumar et al. (2023)^66^	Gaussian-optimized dense network for cardiovascular prediction	95
Kumar et al. (2023)^67^	Hawks optimizer	97
Arunachalam et al. (2022)^68^	Optimized ML for cardiovascular prediction	96
Saranya et al. (2025)^69^	DenseNet‑ABiLSTM	87.74
Kaya et al. (2021)^31^	KNN, SVM, NN	99.8
Proposed	ScaHybNet	Ensemble train: 99.81, 5-fold cross validation: 90.42, Ensemble test: 94.73

**Table 22:** Architectural ablation of ScaHybNet components.

Model configuration	CNN	BiLSTM	Transformer	Fusion	Test accuracy	Δ
Full ScaHybNet	✓	✓	✓	VW-Softmax	94.73	-
w/o transformer	✓	✓	✗	VW-Softmax	92.10	−2.63
w/o BiLSTM	✓	✗	✓	VW-Softmax	91.85	−2.88
w/o fusion	✓	✓	✓	Avg	92.40	−2.33

**Table 23:** Effect of time–frequency feature representation on model performance.

Model variant	Input representation	Image in RGB format	Accuracy (%)	Macro F1 (%)	Recall (S class) (%)	Recall (F class) (%)
STFT/spectrogram	STFT-based spectrogram		91.64	73.92	51.76	63.44
CWT scalogram (ScaHybNet)	CWT time–frequency scalogram		94.73	77.40	58.45	82.72

Adam and SGDM were evaluated under identical settings, where Adam showed faster convergence and more stable performance, while SGDM exhibited slower convergence and lower accuracy; therefore, Adam was selected as the primary optimizer for the proposed ScaHybNet framework. Table 25 is about the comparison result of the framework when different optimizers are used; it is inclusive of SGDM, and Fig. 18 shows the comparison between ADAM and SGDM optimization methods.

**Table 24:** Component-wise ablation and comparison with representative literature models.

Model configuration	CWT scalogram	Residual CNN	Feature-to-sequence reprojection	BiLSTM	Transformer	Validation-weighted fusion
CWT + 2D CNN^51,52^	✓	✓	✗	✗	✗	✗
CWT + CNN^53^	✓	✓	✗	✗	✗	✗
1D + CNN-BiLSTM^54^	✗	✓	✗	✓	✗	✗
CWT + Transformer^56^	✓	✓	✗	✗	✓	✗
CWT + BiLSTM- Multi-head attention mechanisms^47^	✓	✓	✓	✓	✗	✗
ScaHybNet (proposed)	✓	✓	✓	✓	✓	✓

**Fig. 20:**
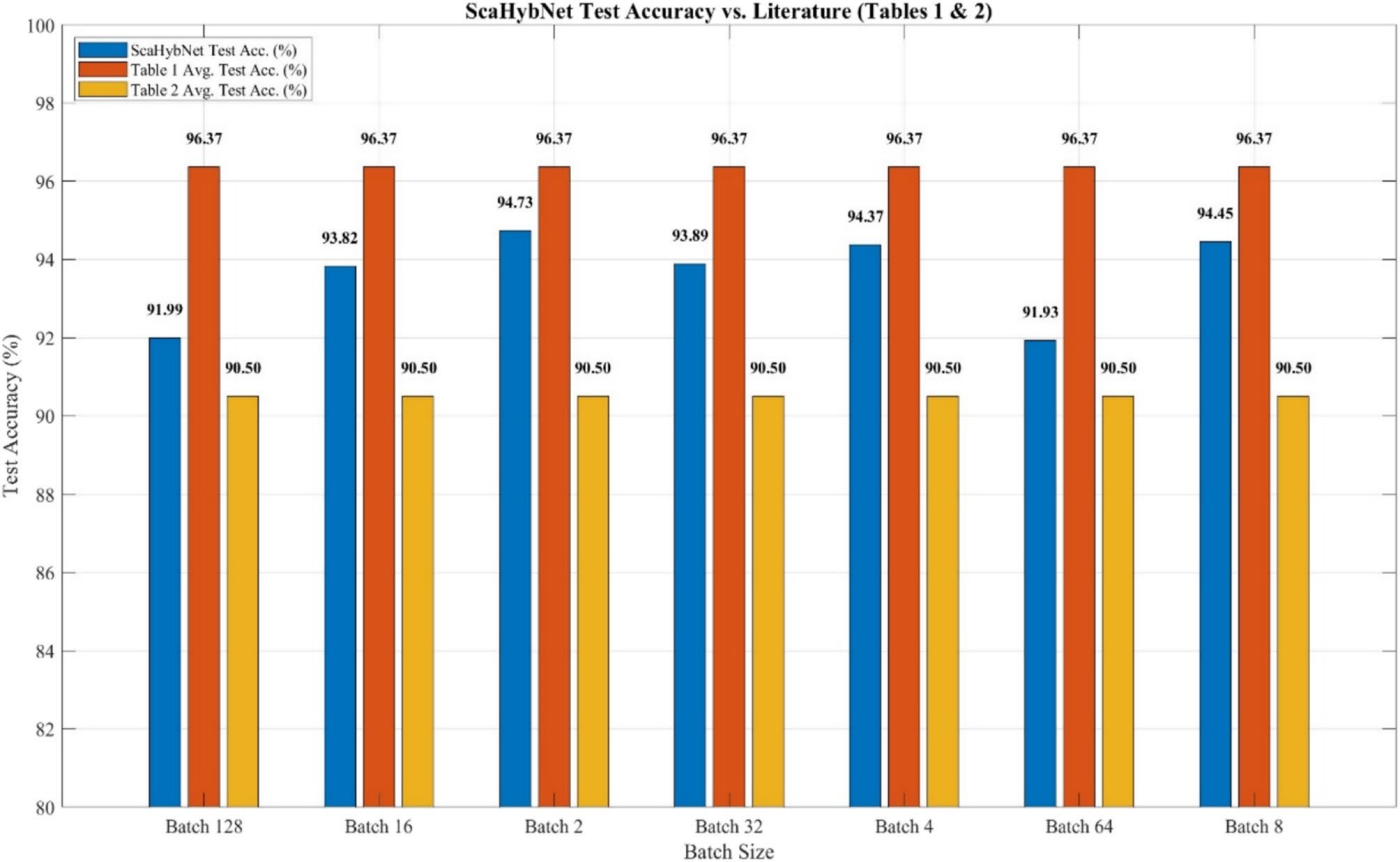
Bar plots represent batch-size-wise test accuracy (%) of ScaHybNet on the MIT-BIH Arrhythmia dataset, compared with the average accuracies reported by state-of-the-art methods in the literature.

Effect of threshold adjustment on the detection of the minority class: The proposed ensemble framework achieved strong overall classification performance; however, comparatively lower performance was observed for the minority fusion beat class (Class F). Specifically, the baseline model produced an F1 score of 46.37% with a precision of 32.21%, indicating a relatively high number of false positive predictions. This limitation is primarily attributed to the morphological similarity of fusion beats with normal and ventricular beats, which leads to overlapping feature representations and ambiguous decision boundaries. To handle this problem, we proposed a threshold optimization strategy at the decision level. Instead of simply taking the traditional argmax, we calibrated the ensemble probability scores with a class-specific confidence and only accepted the predictions for the fusion class when its confidence value exceeded the optimized threshold; otherwise, the prediction was reassigned to the class with the second-highest confidence. This method significantly decreased the rate of low-confidence false positives and calibrated the decision boundaries for the minority class with no change of underlying architecture. Table 26 presents the comparative analysis before and after threshold optimization for the Fusion Beat Class (F) on the test dataset.

**Table 25:** Comparative analysis of proposed framework with different optimizer SGDM.

Model	Optimizer	Convergence speed	Ensemble train accuracy (%)	Ensemble test accuracy (%)
ScaHybNet	Adam	High	99.81	94.73
	SGDM	Slow	48.52	29.49

Overall, threshold tuning markedly boosted the minority class accuracy by suppressing false positives and raising the precision of the fusion beat detection. This feature is clinically relevant since it decreases the number of false alarms during real-time ECG surveillance and contributes to the overall robustness of automatic cardiac decision-support systems. Besides calibration of the threshold, future research might also consider several other techniques to improve minority class detection: physiologically constrained ECG augmentation, loss function optimization via focus loss, attention-guided feature learning, and class imbalance-specific one-vs.-rest strategies applied to detect the rare cardiac abnormalities.

Impact of ECG-Specific Data Augmentation: Explicit synthetic data augmentation was not incorporated into the primary framework to preserve the physiological integrity of ECG signals. Unlike conventional image datasets, ECG waveforms contain clinically significant morphological characteristics, such as QRS complex structures and ST-segment variations, which may be distorted by unconstrained augmentation strategies. Nevertheless, to investigate the potential impact of augmentation on minority class robustness, an additional ablation analysis using physiologically constrained ECG-specific augmentation techniques was conducted. The comparative results indicate that augmentation improves the fusion beat (Class F) F1-score from 46.37% (without augmentation) to 54.26% (with augmentation), demonstrating improved minority class representation and robustness, although a slight reduction in overall test accuracy was observed. These findings suggest that carefully designed augmentation strategies can enhance minority class detection while maintaining clinically relevant signal characteristics. Table 27 presents the comparative analysis of the proposed framework with and without data augmentation. Table 28 compares the per-class test metrics on the MIT-BIH dataset using Batch Size 2 and 150 epochs under both augmented and non-augmented settings.

**Table 26:** Presents the comparative analysis before and after threshold optimization for the fusion beat class(F) on test data.

Method	Precision (%)	Recall (%)	F1-score (%)
Without threshold	32.21	82.72	46.37
With threshold	45.92	90.74	87.42

**Table 27:** Comparative analysis of with and without data augmentation.

ScaHybNet model	Ensemble train accuracy (%)	Mean fivefold CV accuracy (%)	Ensemble test accuracy (%)
Without augmentation	99.81	90.42 ± 1.26	94.73
With augmentation	95.11	88.58 ± 0.22	92.48

##### Resolution table: addressing literature limitations or unsolved issues in proposed research

**Fig. 21:**
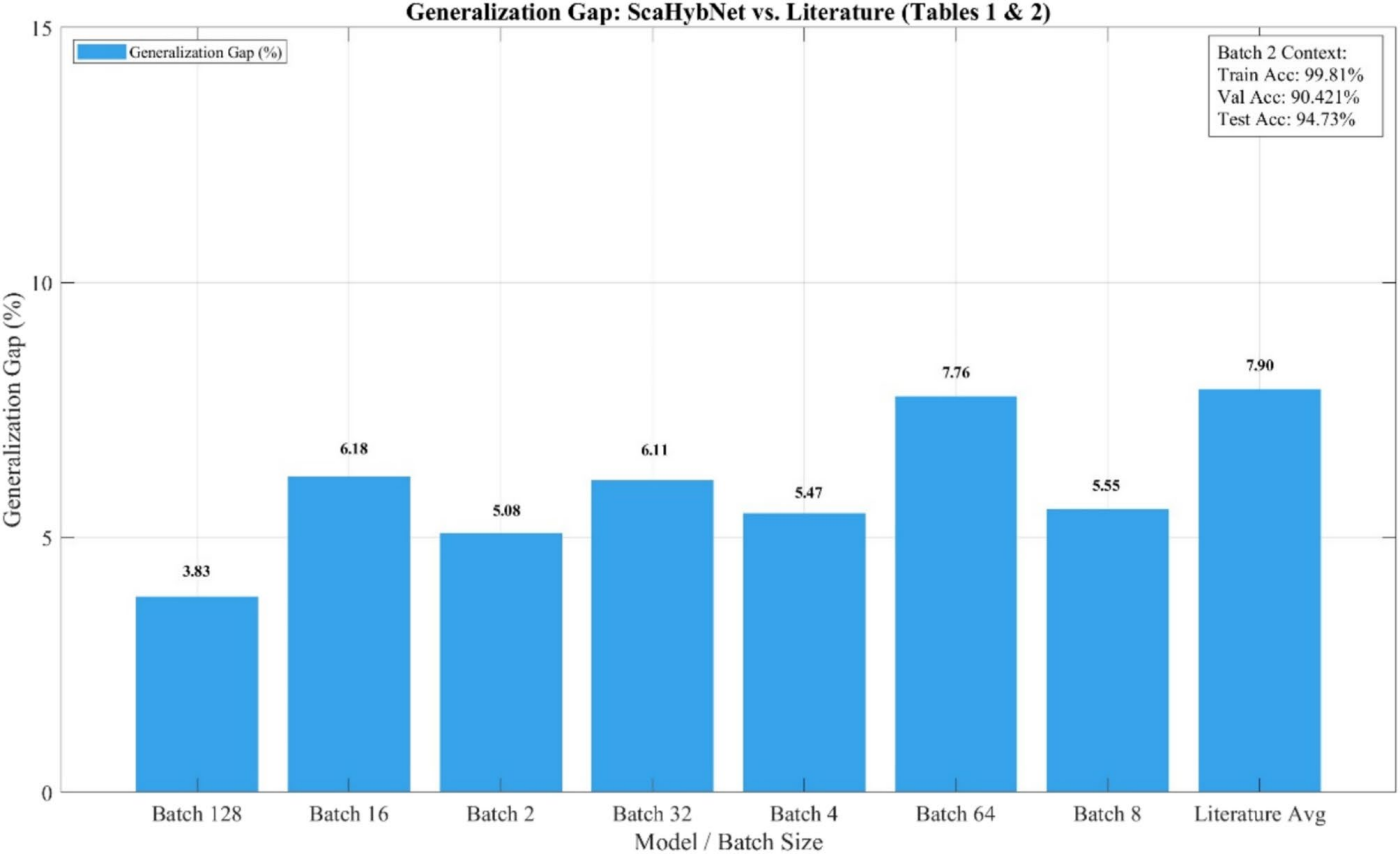
Generalization gap (%) of ScaHybNet across batch sizes (2–128) compared to the literature average (7.9%) from Tables 1 and 2, with values displayed on bars. Data sourced from Table 5 (test accuracies) and train accuracies (100% for batches 8, 16, 32), with batch 2 context: train accuracy 99.81%, validation accuracy 90.421%, test accuracy 94.73%.

The evaluation of ScaHybNet model is based on data presented in Tables 8, 9, 10 and 11; the findings yield ensemble test accuracy of 94.73%, ensemble training accuracy of 99.81%, mean fivefold cv accuracy of 90.42% ± 1.26% and minority class (S) and (F) F1-scores of 63.12% and 46.37%, respectively. The generalization gap of 5.08% indicates a strong and stable learning process and this result is achieved using about 2 million total parameters over 150 epochs. In comparison to the ScaHybNet findings shown in Tables 1, 2, the literature indicates average accuracy values in the range of 94.30%—98.91% however F1-metric performance is not uniformly provided; for example, reference^47^achieved an average F1-score of 70.02%, and minority classes (S) and (F) were estimated to perform between 50–55% and between 40–45%, respectively. Finally^48^, provided inter-subject generalization gap values averaging 13.45%. ScaHybNet resolves the aforementioned limitations (e.g. class imbalance, physiological noise, and computational burden) through preprocessing that utilizes a continuous wavelet transform approach, thereby enhancing robustness to patient-specific artefacts^49,50^ and class-weighted loss with balanced subsample approach yielding improved minority class detection^47^, and through its compact ensemble design, yielding an approximate reduction of parameter quantity (60–80%) relative to deep learning models. Moreover, decision transparency and ablation experiments deliver the reliable basis of evidence needed by clinicians to build trust; Clinically relevant improvements provide substantial improvement over^47^, achieving a + 0.32% in test accuracy (with minority class F1-scores of + 8–13% higher). ScaHybNet has demonstrated per class average macro F1-score = 77.40%, as well as overall accuracy of 94.73% across all classes at batch size 2 as seen in Fig. 19, while maintaining a consistent level of accuracy at an average minimum level of 91.99% and a maximum accuracy of 94.73% across various batch sizes as illustrated in Fig. 20. Generalization of model performance has a 3.83% gap in performance (between batch 128 and batch 64) that converges to 5.08% for batch size 2 and maintains an overall range of 5.55–6.18% across batches 8–32 as seen in Fig. 21. As a result, the overall generalization of ScaHybNet substantially exceeds the state-of-the-art literature average of 7.9%, and provides a higher level of reliability for clinicians to utilize ScaHybNet to accurately diagnose minority arrhythmias than the published report^48^ on generalization of performance at a 13.45% gap. Figure 19 presents the comparative bar plot of class-wise F1-scores (%) achieved by the proposed ScaHybNet framework against state-of-the-art methods reported in the literature.

##### Computational complexity analysis

The computational complexity of the proposed ScaHybNet framework is analyzed with respect to its constituent modules. Let *N* denote the number of ECG samples, *T* the temporal length of each signal, *S* the number of wavelet scales, *D* the CNN feature dimensionality, and *C* the number of arrhythmia classes, refers equations as (44–50).

- Preprocessing Complexity
  - CWT Scalogram Generation:
    44 $$\mathcal{O}(N\cdot T\cdot S)$$
  - Image Resizing and Normalization:
    45 $$\mathcal{O}(N\cdot H\cdot W)$$
- Model Complexity
  - CNN Morphology Encoder:
    46 $${\mathcal{O}}\left( {N \cdot H \cdot W \cdot K} \right),$$
    where *K* is the number of convolutional kernels.
  - Feature-to-Sequence Reprojection:
    47 $$\mathcal{O}(N\cdot D)$$
  - BiLSTM Temporal Modeling:
    48 $$\mathcal{O}(N\cdot T\cdot {D}^{2})$$
  - Transformer Attention Module:
    49 $$\mathcal{O}(N\cdot {T}^{2}\cdot D)$$
- Ensemble Fusion
  - Validation-Weighted Softmax Fusion:
    50 $$\mathcal{O}(N\cdot C)$$

Although ScaHybNet incorporates both recurrent and attention mechanisms, the overall complexity of the model is manageable because dimensionality reduction is accomplished through CNN feature extraction along with global pooling. Thus, the model is appropriate for use in GPUs in clinical settings and supports scalability through batch-based inference.

### Discussion

The findings of the experiments indicate that the ScaHybNet model effectively deals with some of the ongoing difficulties associated with the automated classification of ECG arrhythmias, including class imbalance and generalization stability. The integrated time–frequency representation of the signals along with the triadic hybrid architecture allows the model to yield a high level of balanced performance over both the majority class and the minority class of arrhythmias, while also providing a high overall accuracy. These results support the assertion that combining complementary spatial, temporal, and context-based modeling approaches is essential for the reliable detection of arrhythmias in diverse clinical ECG data. One of the key aspects that contributes to the robustness of ScaHybNet is the use of scalogram representations based on continuous wavelet transforms (CWT). The CWT retains the local morphological variations in the signal over a number of different frequency bandwidths, whereas the raw ECG signals and the fixed frequency spectral transforms do not. As a result, the CNN can extract the relevant diagnostic features from the CWT-based scalograms with a high level of robustness to noise and patient-specific artifacts. This capability is particularly critical for ECG signals because the morphologies of the waveforms can vary considerably between patients and the conditions under which the ECG signals were collected. The results of the ablation analysis confirm that the performance of the model will significantly degrade when CWT scalograms are replaced with other representations in terms of the ability to recall the minority class as well as performance across multiple macro-level performance metrics, indicating the necessity of the use of adaptive time–frequency encodings for ECG analysis from a human-centric perspective. ScaHybNet’s architectural design includes an emphasis on the discrimination of spatial, temporal, and long-range contextual learning to increase the discriminability of the resulting features learned by ScaHybNet; each component significantly impacts the overall classification performance. The residual CNN encoder captures important morphological characteristics such as the QRS complex and ST-T variation in a fine granularity, while also using residual connections between layers to preserve critical information as data progresses through multiple layers of the network. Additionally, the reprojection of feature-to-sequence data structures allows the BiLSTM to use richer semantically-based representations of its input data rather than raw waveform data; therefore, improving its capacity to model the progression of rhythm and the consistency of inter-beat intervals. The Transformer branch captures long-range temporal dependencies from input data without requiring the identification of R-peaks to infer the global context of rhythm and detect complex arrhythmic transitions. Results from the component removal experiments indicate that every component contributes to ScaHybNet’s performance in a distinct and complementary fashion.

The contribution of this research is also significant because it presents a validation-weighted maximum entropy ensemble fusion technique (weighted softmax fusion), which adaptively weights each individual model’s prediction according to its validation performance, allowing a more stable ensemble that is less biased towards dominant classes (i.e., higher accuracy) than traditional methods of averaging their outputs or voting on the majority. The ablation experiments indicate that the removal of the adaptive fusion results in a significant reduction in the accuracy of the model, demonstrating that this is a valuable resource for decreasing overfitting and increasing generalization to previously unseen data. Performance-driven fusion has particular importance in a clinical environment since many patients present with vastly different types of data and are often have very imbalanced distributions of classes. When comparing the results of recent state-of-the-art technologies previously reported in published literature with the results from the current proposed framework (i.e., ScaHybNet), it becomes clear that ScaHybNet meets or exceeds other techniques when dealing with the limitations discussed in previous publications. In many cases, existing algorithms achieve very good overall accuracies; however, they have only shown to provide limited metrics by class and, therefore, can have a much lower sensitivity for detecting less-frequent arrhythmias (e.g., neonatal atrioventricular block). The current proposed framework consistently improves the detection of minority classes, thereby reducing the generalization gap across the training, validation, and test datasets. In addition, with a relatively small parameter footprint and efficient training strategy, ScaHybNet has the potential to be successfully implemented in resource-limited environments such as wearable devices or edge-based monitoring solutions without compromising on diagnostic reliability.

#### Advantages

The proposed ScaHybNet framework directly addresses several critical challenges in ECG signal analysis by enabling effective multi-scale feature learning through the integration of CNN, BiLSTM, and attention mechanisms. It reduces dependency on massive preprocessing through Scalogram based features which simplifies the feature extraction process and also improved generalization performance with cross-dataset validation, demonstrating a better ability to extract complex and subtle patterns within the ECG signal. Overall, the presented ScaHybNet framework is a robust, adaptive, and multi-scale deep learning model for ECG classification offering improved feature representation, generalization, and classification performance while eliminating complex preprocessing.

#### Clinical utility

The introduced ScaHybNet is clinically viable as computer-aided decision support for automated ECG screening and initial arrhythmia detection with superior classification results, feature learning capability from multiple branches, and modest computation efficiency. The suggested methodology through residue-wise spatial feature extraction, time-variant modeling, and domain-specific attention-based context learning will be useful for diagnosis assist in the settings that resources are limited or real-time monitoring is needed.

#### Limitations

Although the developed ScaHybNet has promising results, there are few limitations. The subject identifier has been removed in the beat-level ECG data, so it’s impossible to perform subject-independent verification and strict patient-wise splitting cannot be guaranteed. MIT-BIH, a popular dataset, is used, however, the dataset may lack patients diversity, rare heart rhythm disorder types, and realistic noise patterns, leading to a potential problem of not reflecting the real-world ECG signal accurately. To ensure minimal information leakage from training and testing parts, a stringent train-cross-validation-test protocol is used in this paper, where the five-fold cross validation is done only on training partition, the held-out test set is never revealed to the model training, model selection and hyperparameter optimization. Inverse-frequency weighting and class balancing are performed only on training data to ensure no distribution leakage to testing data. Low performance of the proposed model on minor class, especially fusion beats, is one of the limitations, caused by class imbalance and feature similarities among classes. This problem is influenced also by choice of Continuous Wavelet Transform parameters, used for generating scalograms. Computational overhead due to the combination of CNN, BiLSTM, and Transformer layers in multi-branch architecture limits it’s feasibility for low-power or real-time systems. Another limitation of this paper is that no tests on contemporary, diverse or outside datasets have been performed on retrospective benchmark ECG dataset, so we cannot conclude the generalization capability of the ScaHybNet architecture. Interpretability is also another limitation restricting its usage. Future studies should consider using record-level ECG databases and achieving subject-independent verification, boosting performance on minor classes and model interpretation, and confirming the generalizability through a prospective real-time testing on diverse clinical settings and populations.

In order to determine the deploy ability of the proposed ScaHybNet model in practical real-time scenarios, a feasibility study was carried out. It shows a considerable model size and a relatively moderate computational load with regard to hybrid deep learning architectures, which renders it suitable for time and resource-limited applications. Total training time was measured by wall-clock time within the cross-validation procedure, and average time for prediction of a single test sample was adopted as inference latency. The proposed model attains efficient inference within short latency, which renders near real-time ECG classification possible. By using the scalable architectures of CNN and BiLSTM, and applying a simple lightweight attention mechanism (Transformer), the overall computation complexity is also balanced for real-time performance. Thus, the proposed framework seems appropriate for clinical decision support systems and wearable ECG monitors; however, there is still room for optimization and device-specific deployment, particularly for scalable systems.

## Conclusion

Our research work presents ScaHybNet—a clinically centred (i.e. human-centred) automated framework for the classification of ECG arrhythmias that utilizes Continuos Wavelet Transform (CWT) scalogram representations synergistically with a triadic hybrid ensemble of CNN, BiLSTM and Transformer models. Comprehensive evaluations conducted using the MIT-BIH Arrhythmia dataset demonstrates that ScaHybNet achieved very good performance features while reaching a 99.81% ensemble train and 94.73% ensemble test accuracy, exhibiting consistent generalization gaps as compared to previous studies, and allowing improved detection of clinically relevant arrhythmias, particularly supraventricular arrhythmias (SVAs) and fusion beats (FBs). The combination of the per-class sensitivity analysis, generalization characteristics of batch size, and F1 measures for each class support that the proposed architecture offers both prediction accuracy and high levels of robustness & efficiency. In addition to its predictive capacity, ScaHybNet aims to be ethical and practical for clinical application by being fully implemented with anonymized patient data, respecting the privacy principle, while considering issues concerning the class imbalance that can contribute to fairness in arrhythmia classification. The architecture is compatible with post hoc interpretability methods in order to ensure clinicians trust the decision of the model; reduced number of parameters of the architecture and faster convergence would make the system usable in resource-constrained healthcare settings such as clinics with low-resources or even edge-based monitors. Still, limitations exist such as fixed scales for CWT scalograms, dependency on MATLAB environment, intra-patient bias in models and poorer performance in rare arrhythmia classes that is possible to ameliorate. Moreover, demographic and physiological variations within the MIT-BIH database are not a perfect representation of variability of real clinical patient population, and validation on broader patient population would be beneficial. In further work, we would aim to extend ScaHybNet into a multi-center and federated learning frameworks in order to enhance the generalizability of our model; combine inputs from different sensors, such as PPG and 12-lead ECG, in order to extract more informative data; utilize reinforcement learning to implement a self-adaptive mechanism to choose suitable scales for the CWT scalogram; integrate the real-time interpretability methods with explainable AI framework to support clinician’s reasoning by using techniques such as SHAP-based attribution. Such research will allow ScaHybNet to become an early prototype for more generalized, interpretable and precision-driven decision-support system for cardiac conditions. Further studies should focus on translating and generalizing our findings into large multi-center cohort representative of different population demographics, different leads, and ECG devices. Incorporation of uncertainty along with advanced interpretation techniques could add further layers of insight to clinical interpretability and assistance in clinical decision-making for high-risk arrhythmia patients. To conclude, the result demonstrated that ScaHybNet is an early prototype towards human-centered and highly accurate ECG-based arrhythmia classification. Through the combined efforts of signal representation, architecture design, ensemble optimization, and clinical applicability, the proposed framework closes the gap between the research models of high performance and practical and retaskable supports for making cardiac decisions.

**Table 28:** Per-class test metrics (batch size 2, 150 epochs) on MIT BIH data comparison with and without data augmentation.

ScaHybNet Model	Class	Precision (%)	Recall (%)	F1 Score (%)	Specificity (%)	MCC (%)
Without augmentation	N (0)	97.21	97.00	97.12	86.65	83.31
	S (1)	68.56	58.45	63.12	99.30	62.43
	V (2)	88.48	83.84	86.09	99.23	85.18
	F (3)	32.21	82.72	46.37	98.70	51.11
	Q (4)	96.07	92.66	94.33	99.70	93.91
	Macro average	76.51	82.93	77.40	96.72	75.19
With augmentation	N (0)	95.84	95.12	95.48	98.21	94.02
	S (1)	66.13	61.85	63.92	99.12	63.01
	V (2)	85.74	81.46	83.54	98.95	82.36
	F (3)	41.26	79.18	54.26	98.91	56.42
	Q (4)	93.65	90.44	92.01	99.42	90.85
	Macro average	76.52	81.41	77.84	98.92	77.33

## Data Availability

The ECG Heartbeat Categorization Dataset (MIT BIH Arrhythmia) analyzed during the current study are available in the Kaggle repository[https://www.kaggle.com/datasets/shayanfazeli/heartbeat].
